# Epidemic Dynamics of *Vibrio parahaemolyticus* Illness in a Hotspot of Disease Emergence, Galicia, Spain

**DOI:** 10.3201/eid2405.171700

**Published:** 2018-05

**Authors:** Jaime Martinez-Urtaza, Joaquin Trinanes, Michel Abanto, Antonio Lozano-Leon, Jose Llovo-Taboada, Marta Garcia-Campello, Anxela Pousa, Andy Powell, Craig Baker-Austin, Narjol Gonzalez-Escalona

**Affiliations:** Centre for Environment Fisherie,s and Aquaculture Science, Weymouth, Dorset, UK (J. Martinez-Urtaza, A. Powell, C. Baker-Austin);; Universidad de Santiago de Compostela, Santiago de Compostela, Spain (J. Trinanes);; National Oceanic and Atmospheric Administration, Atlantic Oceanographic and Meteorological Laboratory, Miami, Florida, USA (J. Trinanes);; University of Miami, Miami (J. Trinanes); University of La Frontera, Temuco, Chile (M. Abanto);; Laboratory ASMECRUZ, Bueu, Spain (A. Lozano-Leon);; Hospital Clínico Universitario de Santiago de Compostela, Santiago de Compostela (J. Llovo-Taboada);; Complexo Hospitalario de Pontevedra, Pontevedra, Spain (M. Garcia-Campello);; Direccion Xeral de Innovación e Xestión da Saúde Pública, Consellería de Sanidade, Xunta de Galicia, Galicia, Spain (A. Pousa, A. Powell);; US Food and Drug Administration, College Park, Maryland, USA (N. Gonzalez-Escalona)

**Keywords:** epidemiology, bacteria, *Vibrio parahaemolyticus*, seafood safety, gastroenteritis, food safety, whole-genome sequencing, Galicia, Spain, global phylogeny, warming, enteric infections, climate change

## Abstract

Galicia in northwestern Spain has been considered a hotspot for *Vibrio parahaemolyticus* infections. Infections abruptly emerged in 1998 and, over the next 15 years, were associated with large outbreaks caused by strains belonging to a single clone. We report a recent transition in the epidemiologic pattern in which cases throughout the region have been linked to different and unrelated strains. Global genome-wide phylogenetic analysis revealed that most of the pathogenic strains isolated from infections were associated with globally diverse isolates, indicating frequent episodic introductions from disparate and remote sources. Moreover, we identified that the 2 major switches in the epidemic dynamics of *V. parahaemolyticus* in the regions, the emergence of cases and an epidemiologic shift in 2015–2016, were associated with the rise of sea surface temperature in coastal areas of Galicia. This association may represent a fundamental contributing factor in the emergence of illness linked to these introduced pathogenic strains.

Globally, *Vibrio parahaemolyticus* is the leading bacteriological cause of illness associated with seafood consumption. Infections have undergone a global expansion over the last 2 decades; cases have suddenly emerged in areas considered environmentally adverse for these pathogens (*1*–*3*). The causes of this dynamic expansion and emergence in non–disease-endemic areas have remained elusive.

*V. parahaemolyticus* infections are generally rare and sporadic across all of Europe with a single exception: Galicia in northwestern Spain. This region has been considered a hotspot for *Vibrio* infections and an anomaly within the epidemiologic context of *V. parahaemolyticus* in Europe; reccurring cases of foodborne vibriosis (*4*–*7*) and outbreaks (*7*,*8*) have been reported regularly since the late 1990s. Infections associated with *V. parahaemolyticus* in Galicia were characterized by sudden outbreaks of illness typically associated with a single genetic variant of the pathogen (*6*,*8*). The first sign of change in this epidemiologic pattern was observed in 2012 when 3 different and genetically unrelated strains of *V. parahaemolyticus* were identified during a large outbreak in Galicia (*5*,*9*). Since then, a clear transition in the epidemiology of this pathogen has been observed; sporadic cases scattered throughout the region have been caused by different and unrelated strains and typically associated with the consumption of locally produced shellfish.

We applied whole-genome sequencing for a comprehensive and high-resolution insight into pathogenic populations identified in clinical sources associated with the major episodes of illness in Galicia over the past 20 years. We performed phylogenetic analysis to identify the population structure and potential sources of the clinical strains identified in Galicia. We also conducted a parallel exploration of the variability of environmental conditions in the region to investigate the existence of other factors contributing to the emergence of illness linked to these in this particular area.

## Materials and Methods

### Bacterial Strains and DNA Extraction

We analyzed 18 isolates derived from clinical sources collected over the course of the different outbreaks in Galicia (Table). All the strains isolated from infections in Galicia were characteristically *tdh* positive and *trh* negative, the only exceptions being the strains belonging to sequence type (ST) 36 isolated during the 2012 outbreak, which were positive for both haemolysin genes. Additionally, we included 14 isolates obtained from environmental sources (shellfish and zooplankton) during 2003–2007 to analyze the potential connection between the clinical pathogenic populations and local marine environmental sources. Finally, we added 4 clinical strains reported in the United Kingdom associated with human infections since the 1970s to the study to explore possible connections between pathogenic populations within Europe, along with another 6 environmental strains from the United Kingdom isolated in 2014.

**Table:** Characteristics of *Vibrio parahaemolyticus* strains sequenced and analyzed for study of epidemic dynamics, 1998–2016*

Strain	CFSAN no.	Year	Source	ST	*tdh*	*trh*	Accession no.†	Reference
Strains identified in Spain								
30824	CFSAN018753	1999	Clinical	ST17	+	–	LHAV00000000	(*6*)
428–00	CFSAN018752	1998	Clinical	ST17	+	–	LHAU00000000	(*6*)
9808–1	CFSAN018754	2004	Clinical	ST3	+	–	LHAW00000000	(*8*)
118	CFSAN045068	2015	Clinical	ST1031	+	–	SRR5163839	This study
119	CFSAN045069	2015	Clinical	ST1031	+	–	SRR5163836	This study
113477	CFSAN045070	2015	Clinical	ST327	+	–	SRR5163834	This study
AMC 317	CFSAN056086	2016	Clinical	ST3	+	–	SRR5163849	This study
AMC 325	CFSAN056088	2016	Clinical	ST1031	+	–	SRR5163835	This study
G25	CFSAN022330	2012	Clinical	ST36	+	+	LHRR00000000	This study
G30	CFSAN022331	2012	Clinical	ST36	+	+	LHRS00000000	This study
G31	CFSAN022332	2012	Clinical	ST36	+	+	LHRT00000000	This study
G32	CFSAN022337	2012	Clinical	ST1032	–	+	SRR5163840	This study
G33	CFSAN022333	2012	Clinical	ST1031	–	+	SRR5163848	This study
G35	CFSAN022336	2012	Clinical	ST36	+	+	LHRW00000000	This study
G36	CFSAN022335	2012	Clinical	ST36	+	+	LHRV00000000	This study
G37	CFSAN022334	2012	Clinical	ST36	+	+	LHRU00000000	This study
N310	CFSAN053627	2016	Clinical	ST327	+	–	SRR5163837	This study
N314	CFSAN053626	2016	Clinical	ST3	+	–	SRR5163842	This study
OAG100	CFSAN025076	2007	Shellfish	ST1121	+	+	SRR5163838	This study
OAG95	CFSAN025079	2007	Shellfish	NA	–	+	SRS1912582	This study
OAG99	CFSAN025078	2007	Shellfish	ST1121	+	+	SRS1912583	This study
OJL90	CFSAN029659	2007	Shellfish	ST331	+	–	SRR5163850	This study
PH157	CFSAN025074	2006	Zooplankton	ST331	NA	NA	SRR5163833	This study
PQ110	CFSAN029660	2006	Zooplankton	ST79	–	+	SRR5163846	This study
PY194	CFSAN025072	2007	Zooplankton	ST199	+	+	SRR5163847	This study
PY233	CFSAN025077	2006	Zooplankton	ST169	–	–	SRS1912575	This study
PY350	CFSAN025073	2006	Zooplankton	ST1032	–	+	SRR5163841	This study
PY452	CFSAN025075	2007	Zooplankton	ST1032	+	–	SRS1912576	This study
PY456	CFSAN025071	2006	Zooplankton	ST1032	–	+	SRR5163847	This study
UCM-V441	CFSAN018755	2002	Shellfish	ST52	–	–	LHAX00000000	This study
UCM-V493	NA	2002	Sediment	ST471	–	–	CP007005, CP007004	This study
UCM-V586	CFSAN018756	2003	Shellfish	NA	–	–	LHAY00000000	This study
Strains identified in the United Kingdom								
14-1072-D-VP	CFSAN029647	2014	Shellfish	ST1159	–	+	SRR5639920	This study
14-1073-H-VP	CFSAN029643	2014	Shellfish	ST1159	–	+	SRR5639916	This study
14-1498-F-VP	CFSAN029646	2014	Shellfish	ST1158	–	+	SRR5639919	This study
14-1499-VP	CFSAN029645	2014	Shellfish	ST1157	+	+	SRR5639914	This study
14-559-B-VP	CFSAN029644	2014	Shellfish	ST1159	–	+	SRR5639913	This study
14-692-A-1-VP	CFSAN029642	2014	Shellfish	ST1159	–	+	SRR5639915	This study
V12-024	CFSAN029651	2014	Clinical	ST3	+	–	SRR5639912	This study
V05-002	CFSAN029650	1972	Clinical	ST331	+	–	SRR5639911	This study
V06-002	CFSAN029649	1980	Clinical	ST17	+	–	SRR5639918	This study
F3305-VP	CFSAN029648	2005	Clinical	ST262	+	–	SRR5639917	This study

*CFSAN, Center for Food Safety and Applied Nutrition; NA, not applicable; SRA, Sequence Read Archive; ST, sequence type; +, positive; –, negative. †National Center for Biotechnology Information Assembly or Sequence Read Archive database.

### Genome Sequencing and Sequence Processing

We performed genomic DNA extraction of the 42 strains from overnight cultures using the DNeasy Blood & Tissue Kit (QIAGEN, Hilden, Germany). We sequenced the genomes of all 42 strains using MiSeq (Illumina, San Diego, CA, USA) with a minimum coverage of 40–120-fold. We prepared libraries with the Nextera XT DNA sample preparation kit (Illumina) and de novo assembled whole-genome sequence contigs for each strain by using CLC Genomics Workbench version 7.5.1 (QIAGEN, Valencia, CA, USA).

### Global Collection of *V. parahaemolyticus* Genomes

We initially investigated the position of the strains from Spain and the United Kingdom on the global phylogeny of *V. parahaemolyticus* using all the available *V. parahaemolyticus* genomes worldwide, including 696 genomes obtained from the National Center for Biotechnology Information Assembly and Sequence Read Archive (SRA) databases (Technical Appendix Table) plus the 42 genomes sequenced in our study. We transformed the SRA data to fastq using SRA Toolkit (fastq-dump –split-files –gzip –skip-technical) (https://trace.ncbi.nlm.nih.gov/Traces/sra/sra.cgi?view=toolkit_doc). We performed genome assembly with A5-pipeline (*10*). We performed in silico inference of MLST profiles and STs using MLST software (https://github.com/tseemann/mlst), which infers STs using the public MLST scheme for *V. parahaemolyticus* based on 7 housekeeping genes (https://pubmlst.org/vparahaemolyticus/).

### Single-Nucleotide Polymorphism Calling and Phylogenetic Inference

Single-nucleotide polymorphisms (SNPs) were called using Harvest version 1.0.1 (https://github.com/marbl/harvest) (*11*). We used Parsnp, a component of the Harvest suite, to align the assembled genomes and define the core genome. We identified SNPs for both chromosomes by Parsnp in the multi-alignments; we used filtered and reliable core-genome SNPs to construct a core genome maximum-likelihood phylogenetic tree.

### Analysis of Sea Surface Temperature Trend Off the Coast of Galicia, Northwest of Spain

We estimated trend in the mean values of sea surface temperature (SST) using daily SST data from a coastal area limited by the coordinates 42°N–43°N and 8.5°W–9.5°W. The mean SST data come from the Optimum Interpolation SST 1/4° daily dataset (OISST), which extends from September 1981 to the present and is distributed by NOAA/NCEI. This dataset combines satellite retrievals and in situ SST data from ships and buoys. We use these analyzed fields to estimate the trends in the region of interest, detect possible regime shifts, and study the habitat suitability of *Vibrio* spp. in the region. We investigated regime shift, defined as rapid reorganizations of ecosystems from one relatively stable state to another, using Sequential Regime Shift Detection Software version 3.2 (http://www.beringclimate.noaa.gov/regimes/). This program detects statistically significant shifts in the mean level and magnitude of fluctuations in time series taking the autocorrelation into account (*12*). The program detects shifts in the mean level of SSTs. The method is based on a sequential *t*-test that can signal a possibility of a regime shift. We used the default significance level of 0.1 that represents the level at which the null hypothesis that the mean values of the 2 regimes are equal is rejected by the *t*-test.

### Nucleotide Sequence Accession Numbers

The draft genome sequences of all 44 *V. parahaemolyticus* strains from our study are available in GenBank. Accession numbers are provided in the Table.

## Results and Discussion

**Figure 1:**
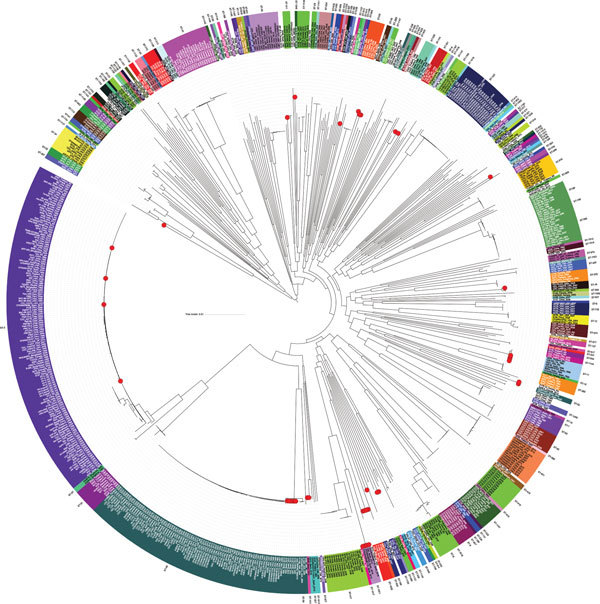
Phylogenetic reconstruction of *Vibrio parahaemolyticus* based on 738 available genomes. Red dots indicate isolates from Spain collected over the past 20 years from clinical settings and environmental sources. Colors represent sequence types, and areas without color correspond to undetermined sequence types. Scale bars represent nucleotide substitutions per site.

**Figure 2:**
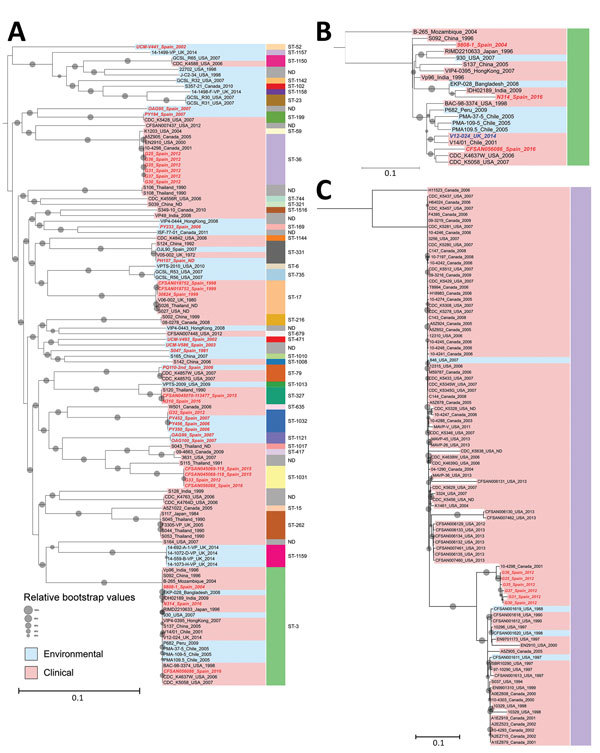
Phylogeny of *Vibrio parahaemolyticus* isolates from Galicia, Spain. A) Phylogenetic inference of the 42 genomes from Spain identified in this study (red text) along with all other genomes identified in the same clusters by the global phylogeny with their corresponding sequence types (STs). B) Phylogenetic tree of genomes belonging to ST3 (pandemic clone). C) Phylogenetic tree of genomes included in ST36 in the global phylogeny. Gray dots indicate bootstrap values supporting the nodes; dot sizes indicate 80% (smallest) to 100% (largest). Values <80% are not shown. Scale bars represent nucleotide substitutions per site. ND, not determined.

Analysis of the 738 *V. parahaemolyticus* genomes resulted in a core genome alignment of 292,750 bp containing 12,399 SNPs. Positions of the Spanish strains in the global phylogeny (Figure 1) revealed a complex epidemiologic scenario with the existence of multiple, highly diverse genomic variants of strains associated with infections in the region. Moreover, we identified 12 different STs among clinical strains isolated over the past 2 decades (Figures 1, 2). We selected the genomes that clustered together with the genomes from Spain (115 genomes) and included them in a high-resolution phylogenetic reconstruction (Figure 2). The basis for the reconstruction was a core genome alignment of 3,049,195 bp containing 202,859 SNPs.

The original strain that was isolated over the course of the earliest documented large outbreak in Galicia in 1998–1999 (ST17), which pulsed-field gel electrophoresis (PFGE) subsequently reported as a new local clone (*6*), turned out to be closely related to strains previously isolated in Thailand (2006) and the United States (2006) when assessed by whole-genome phylogeny. The clinical strain from the United Kingdom isolated in Maidstone in the late 1970s (National Collection of Type Cultures no. 11344) and reported as genetically related to the Galician strains by PFGE (*6*), was also found to cluster with this group, with a difference of 450 SNPs.

Strains belonging to the so-called Asian pandemic clone (clonal complex [CC] 3) were first reported in Galicia in association with a large outbreak in 2004 (*8*). Epidemiologic analysis of the outbreak traced back the origin of the outbreak to a facility located in the international seaport of A Coruña in Galicia, suggesting that the most probable source of the pandemic strain was the discharge of ballast water carried in ships. No strain from this group was identified in Spain until summer 2016, when we identified 2 strains isolated in July from 2 independent outbreaks in the cities of Silleda and Pontevedra, investigated in 2 different hospitals, as belonging to CC3. Genomic analysis of the strains from Galicia, along with 21 additional genomes belonging to the ST3 strains isolated in other countries, resulted in a core alignment of 3,560,214 bp for the ST3 clade containing 384 SNPs. Whole-genome phylogeny revealed that the 2 strains from Galicia isolated in 2016 belonged to 2 different groups; both are different from the strain identified in Galicia in 2004. We identified strain N314 as part of the Asian group of CC3 and strain CFSAN056086 in the American group. We were able to clearly distinguish these 2 strains from the strains isolated from the 2004 outbreak, which was identified in a separate branch closer to different Asian strains. Of note, while the 2004 outbreak was associated with imported seafood and unsafe food manipulation (*8*), the recent infections caused by CC3 strains were unequivocally associated with local shellfish (razor clams and cockles); compelling evidence of several successful introduction events of these strains into the marine environment of Galicia.

In many ways, the 2012 outbreak in Spain (*5*,*13*) represented a clear change in the epidemic dynamics of *V. parahaemolyticus* in the region. First, this outbreak was the largest reported across Europe linked to local seafood; second, it was the earliest known evidence of a cross-continental spreading of the ST36 clone, which is endemic to the Pacific Northwest (PNW) of the United States and one of the most virulent ST groups (*13*). Genomic analysis of the 92 available genomes of the ST36 isolated from areas of endemicity for this group in the PNW identified a core alignment of 3,310,986 bp comprising 1,596 SNPs. Phylogenetic analysis of the ST36 lineage revealed the existence of 2 different clusters within this group (Figure 2, panel C): a first cluster composed of old strains from Canada isolated before 2005 and the United States before 2000, and a second cluster with modern representatives from the United States and Canada. This particular population structure suggests the existence of a lineage replacement in the PNW coast and western Canada, where only strains belonging to the second cluster were identified from 2005 onward. Surprisingly, we unequivocally identified the strains in the 2012 Galicia outbreak as belonging to the first cluster composed of strains extinct in their original location along the PNW coast, which suggests an early introduction of these strains into waters of Galicia and Europe (*14*). Furthermore, we identified a single strain from Canada as closely related to the genomes from the Galician strains with a minimum difference of 20–22 SNPs in an alignment of 3,310,986 bp, whereas variations among genomes of the ST36 strains from Galicia were 0–19 SNPs. The low level of variation among all the genomes in this clade supports a hypothesis that Galician strains originated in British Columbia, Canada, and were introduced in Galicia sometime after 2001.

A second singularity of the 2012 outbreak, and probably more noteworthy, was the fact that infections from a single outbreak were associated with several unrelated strains of *V. parahaemolyticus*. We identified 2 additional strains different from ST36 from clinical cases over the course of this outbreak, ST1031 and ST1032; both represent novel STs not reported before the 2012 outbreak. Whole-genome phylogeny of these new STs grouped these strains into 2 distinctive clusters. Strain G32, belonging to ST1032, showed a close association with several strains isolated from zooplankton in offshore waters of Galicia in 2006–2007, which could be preliminary evidence of a local origin of these strains introduced by the incursion of offshore oceanic waters. Strain G33, belonging to ST1031, was included in a single group along with strains also associated with local shellfish and isolated over the course of the outbreaks in summers of 2015 and 2016. 

Finally, we identified an additional group of strains belonging to ST327 associated with illnesses over the summer of 2015 and 2016. We included these strains in the same cluster as 1 strain from Thailand isolated in 1990 (Figure 2).

Our genome-wide phylogenetic analysis of pathogenic *V. parahaemolyticus* in northern Spain has provided novel insights into the epidemiology of *V. parahaemolyticus* in nonendemic areas. The primary result is that the study revealed the existence of a complex epidemiologic context characterized by the existence of multiple highly diverse strains, most originating far away, that caused infections associated with locally produced shellfish; this finding could be considered evidence of multiple events of introduction of foreign variants into Galicia. The source of these strains into Galicia is elusive and remains an area of ongoing interest, but we did identify through this study and previous work the 2 proposed mechanisms for dissemination of pathogenic strains: ballast water (*15*–*17*) and zooplankton migration (*18*,*19*). Ballast water has been proposed as a main source of pathogenic *Vibrio* bacteria (*16*) and was suggested as the mechanism of introduction of pandemic strains in the 2004 outbreak (*8*). In addition, we showed new evidence that unequivocally identified environmental transport through offshore zooplankton as one of the routes of introduction of new pathogenic variants via ocean currents (*18*–*20*). However, we cannot rule out the introduction of foreign mollusks into the marine water of Galicia as a possible source of new variants of pathogenic *Vibrio* bacteria from disparate and remote sources because of the magnitude of the shellfish trade in the region; the importation of shellfish from other geographic areas is a common practice to supply the high demand for products. A recent study analyzing the population structure and evolution of the ST36 clone suggests that the importation of clams from the PNW to Spain circa 2000 is the probable source of ST36 strains (*14*). Moreover, 2 other recent studies tracking the routes of introduction of the Manila clam from its original place of distribution in the Indo-Pacific region to Europe has also linked the origin of clam populations introduced in Spain to the PNW of the United States (area of endemicity of ST36 populations) during the importations of clams in the mid- and late 1990s (*21*,*22*). These findings closely correspond with the results shown in our study.

**Figure 3:**
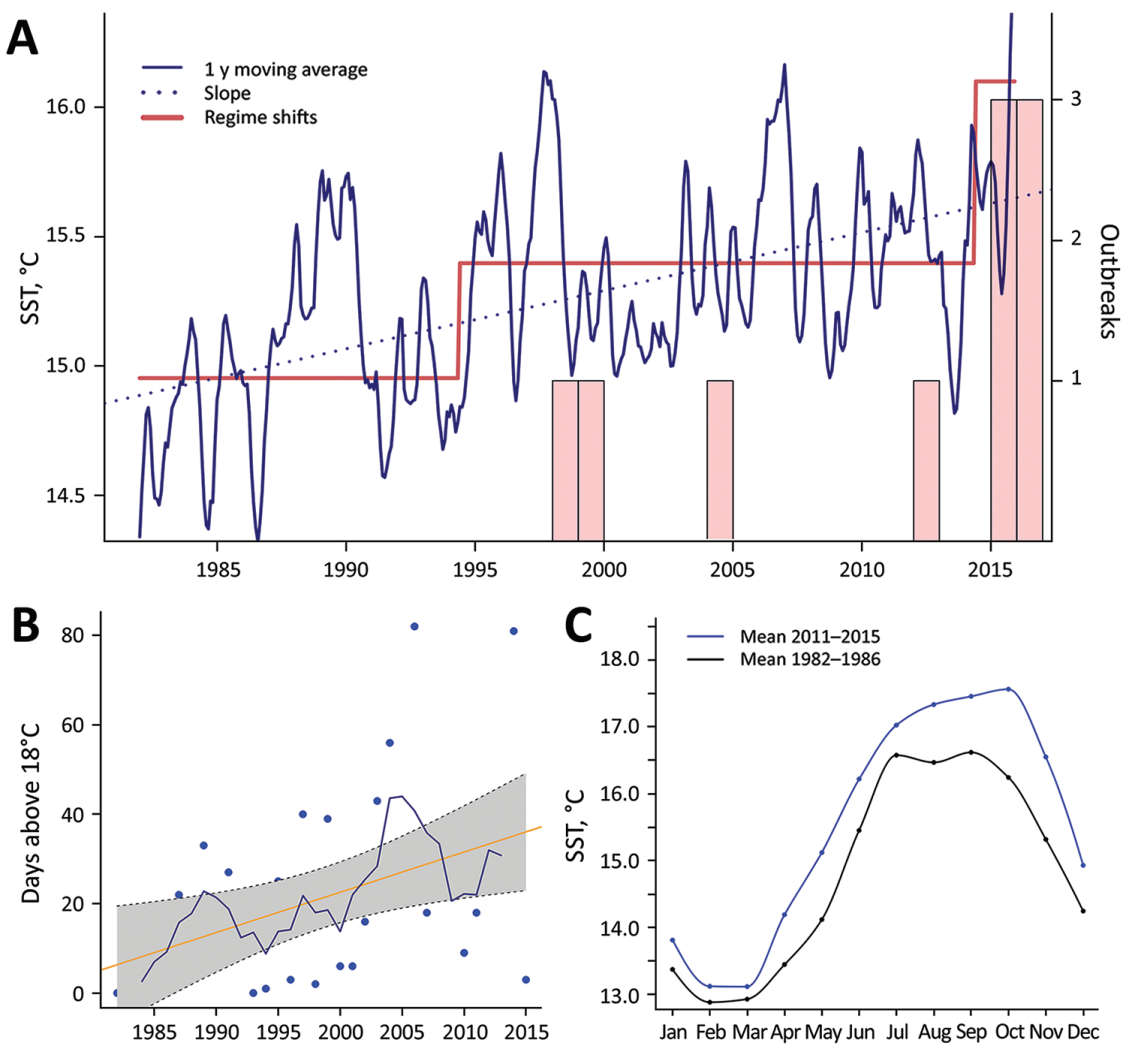
Recent environmental warming trends in Galicia, Spain, 1982–2016. Trends in the mean values of SST were estimated using daily SST data from a coastal area defined by the coordinates 42°–43°N and 8.5°–9.5°W. A) Mean SST records show stepwise changes rather than a linear pattern. Two regime shifts occurred in June 1994 (0.4°C warming) and June 2014 (0.7°C), which correspond with the first emergence of *Vibrio parahaemolyticus* cases and the epidemiologic shifts observed for 2015 and 2016. B) Number of days with SST >18°C (blue dot), 5-year moving average (blue line), and regression line (yellow line); slope is of ≈1 d/y (e.g., gaining 1 d/y). C) Mean SST data for Galicia for 2 periods, demonstrating the generalized warming and expansion of season with favorable conditions for sustaining *Vibrio* organisms in the environment and hence increasing risk of infection. SST, sea surface temperature.

An underlying finding of our study is that the introduction of these highly pathogenic strains into a region is not sufficient by itself to initiate an epidemic; the introduced strains appear established in the area for a substantial period without evidence of associated illness, which suggests additional cofactors in infections and risk. Because seawater temperature has been identified as a critical factor governing the emergence of *Vibrio* diseases (*1*–*3*), we conducted an analysis of historical records of SST in the region. Results from these analyses revealed a significant trend of increased SST in the area, which followed a stepwise and incremental trend, rather than the expected linear change. We identified shifts in both the mean level of fluctuations and the variance of SST time series. We recognized 2 clear shifts of SST with a significant statistical support over the study period: June 1994, an SST increase of 0.4°C; and June 2014, an SST increase of 0.7°C (Figure 3). These 2 shifts in seawater temperature showed a close correspondence with the epidemic dynamics of *V. parahaemolyticus* in the area, showing a period with no infections before the first regime shift, a second period when the first epidemic events were identified, and finally a third period after the last regime shift in 2014, which was concurrent with the change in the epidemiology of *V. parahaemolyticus* we report in this study. Previous data have shown that regime shift warming has led to an increase in prevalence of *Vibrio* bacteria in the environment (*1*,*23*), and epidemiologic studies on the emergence of *Vibrio* infections have identified SSTs >18°C as a critical threshold for triggering infections and substantially increasing the number of reported clinical cases (*2*). Analysis of the number of days with SSTs >18°C in Galicia over the past 36 years identified an increase of 1 day/year (Figure 2, panel B), which resulted in an increase of 35 days for the risk period of *V. parahaemolyticus* infections for the whole period. These results contrast with the situation in some areas of natural endemicity for pathogenic *V. parahaemolyticus*, such as the PNW for the ST36, where seawater temperatures have remained remarkably stable over the past 2 decades and regime shifts have not been detectable. The average annual SST in areas of Puget Sound in the PNW is ≈5°C lower than in Galicia, showing a warming trend almost 2 orders of magnitude smaller and SST values always <18°C, according to the records of the satellite mesoscale SST time series (data not shown).

This study highlights the utility of whole-genome sequencing as a tool to elucidate key features of the transmission and potential sources of pathogenic environmental bacteria such as *Vibrio* spp. The concomitant introduction of foreign *Vibrio* variants with a significant warming trend in the region, coupled with the consumption of locally produced shellfish in the region, may be major contributing factors for the emergence of infections in Galicia. Parallel circumstances may also drive disease emergence in other areas of the world with similar environmental conditions, such as the Pacific Northwest (*24*,*25*) and the Atlantic Northeast (*14*,*26*) in the United States or the south of Chile (*27*). In these areas. The presence of imported *Vibrio* strains has been frequently reported associated with outbreaks, particularly during and after warming events (*28*). These areas represent major contributors to the escalation and global expansion of *V. parahaemolyticus* illnesses associated with the dissemination of the preeminent pathogenic clones of these organisms.

Technical Appendix Information about all genomes used to reconstruct the global phylogeny of *Vibrio parahaemolytus.*
