# Supplementary material for: Epidemic Dynamics of Vibrio parahaemolyticus Illness in a Hotspot of Disease Emergence, Galicia, Spain
Source: Emerg Infect Dis. 2018 May;24(5):852–9. doi: 10.3201/eid2405.171700 (PMC5938774; doi:10.3201/eid2405.171700)
Supplement: Technical Appendix — Information about all genomes used to reconstruct the global phylogeny of Vibrio parahaemolytus. [file 17-1700-Techapp-s1.pdf]

Article DOI: https://doi.org/10.3201/eid2405.171700

Epidemic Dynamics of *Vibrio parahaemolyticus* Illness in a Hotspot of Disease Emergence, Galicia, Spain

Technical Appendix

Technical Appendix Table. Genomes used to reconstruct the global phylogeny of *V. parahaemolytus* with some basic genome assembly statistics*

ID	Alias	Geographical origin	Date	Source	ST	Cluster	No. Contigs	Total length	Largest contig	N50	N75	L50	L75	No. Ns/100 kbp
AWNE01	S002	China	1999	Clinical	ST-216	cluster_1	648	5060436	84656	20468	11712	74	154	0
JZAO01	08-0278	Canada, AB	2008	Clinical	ST-216	cluster_1	188	5206474	855186	402713	195247	5	10	0
MIQU01	GCSL_R30	USA, FL	27/05/07	Oyster	ST-23	cluster_10	131	5132857	517924	294632	120423	7	14	0
MIQV01	GCSL_R31	USA, LA	27/05/07	Oyster	ST-23	cluster_10	132	5126812	517924	294642	119285	7	15	0
AWNF01	S001	Thailand	ND	Clinical	ST-1014	cluster_100	496	5030720	94989	24000	12192	63	137	0
LFZG01	ISF-77-01	Canada	2011	Imported shrimp	ST--	cluster_101	95	5042958	1089609	518506	232242	4	7	0
BAVI01	TUMSAT_H01_S4	Thailand	ND	Shrimp	ST--	cluster_102	70	5213752	506032	271160	174388	7	13	20.74
AWHH01	S173	China	2007	Environmental	ST-289	cluster_103	407	4951244	129114	37764	19317	44	88	0
AWJH01	S118	Japan	1984	Clinical	ST--	cluster_104	722	5134676	54319	12725	7323	116	245	0
SRR3987381	SRR3987381	ND	30/07/2015	Shrimp	ST--	cluster_105	89	5350122	889934	405683	239254	5	9	1.7
JTGS01	4.2548	Canada, ON	2004	Clinical	ST-430	cluster_106	79	5479562	506844	244392	117081	8	16	0
14-1498-F-VP	14-1498-F-VP	UK	18/08/14	Oyster	ST-1158	cluster_107	103	5115518	368763	136595	65153	13	26	0
AWJT01	S105	Japan	1984	Clinical	ST-325	cluster_108	791	5125425	83099	14631	7337	106	224	0
MIQW01	GCSL_R32	USA, LA	30/05/07	Oyster	ST-1142	cluster_109	105	5059594	509644	186217	93747	9	18	0
MIRU01	GCSL_R95	Canada, PEI	31/07/07	Oyster	ST-1151	cluster_11	103	5123173	650220	431216	179648	5	10	0
MIRW01	GCSL_R98	Canada, PEI	31/07/07	Oyster	ST-1151	cluster_11	102	5125449	773349	413198	179377	5	9	0
MIRX01	GCSL_R99	Canada, PEI	31/07/07	Oyster	ST-1151	cluster_11	96	5120561	652163	349147	179613	5	10	0
MIRY01	GCSL_R108	Canada, PEI	31/07/07	Oyster	ST-1151	cluster_11	93	5156159	773296	348887	179346	5	9	0
MIRZ01	GCSL_R109	Canada, PEI	31/07/07	Oyster	ST-1151	cluster_11	93	5124297	773349	349026	178579	5	10	0
MISA01	GCSL_R110	Canada, PEI	31/07/07	Oyster	ST-1151	cluster_11	96	5123801	773349	413181	179377	5	9	0.02
SRR1118657	CDC_K4857G	USA, HI	28/01/07	Stool	ST-1151	cluster_11	89	6033926	699467	378987	178434	6	12	3.52
SRR1118658	CDC_K4842	USA, MD	16/10/06	Stool	ST-1151	cluster_11	95	5127304	607796	409649	179686	5	11	1.78
SRR1118660	CDC_K4764D	USA, VA	13/10/06	Stool	ST-1151	cluster_11	79	5123974	607785	413170	180230	5	10	2.5
SRR1118661	CDC_K4762	USA, VA	15/08/06	Other	ST-1151	cluster_11	107	6024061	564945	270657	142339	8	16	3.23

ID	Alias	Geographical origin	Date	Source	ST	Cluster	No. Contigs	Total length	Largest contig	N50	N75	L50	L75	No. Ns/100 kbp
MISF01	GCSL_R131	USA, FL	01/10/07	Oyster	ST-1141	cluster_110	132	5140538	559015	313100	159145	6	12	0
JTGT01	9.5357	Canada, NB	2009	Clinical	ST-633	cluster_111	688	5367487	877995	387375	193012	4	8	0
AWJY01	S097	China	1992	Clinical	ST--	cluster_112	531	5024814	97359	23502	11394	66	146	0
JOKT01	13-028/A2	Vietnam	2013	Shrimp	ST-1112	cluster_113	213	4943329	461307	143936	86736	10	21	0
BAVK01	TUMSAT_H10_S6	Thailand	ND	Shrimp	ST-977	cluster_114	64	5213844	910293	329189	247856	5	10	23.73
LIRT01	ISF-94-1	Canada	2011	Imported shrimp	ST--	cluster_115	60	5056993	1293058	407861	240825	4	7	0
MISG01	GCSL_R135	USA, SC	21/11/07	Oyster	ST-741	cluster_116	141	5066023	755387	324832	174909	5	10	0
MISE01	GCSL_R129	USA, FL	01/10/07	Oyster	ST-1153	cluster_117	114	4897383	402460	227454	119393	8	15	0
MIRJ01	GCSL_R60	USA, ME	23/07/07	Oyster	ST-1135	cluster_118	126	5264840	647399	256849	142996	6	13	0
JTGR01	T9109	Canada, BC	2007	Clinical	ST-634	cluster_119	202	5112443	813522	484812	196461	5	9	0
JNUO02	CFSAN007454	USA, MD	2010	Oyster	ST-676	cluster_12	36	5017786	799796	445027	266184	5	8	0
LFZD01	HS-13-1	Canada	2014	Clam	ST-676	cluster_12	106	5098325	799855	401486	195874	5	9	0
MIRB01	GCSL_R51	USA, AL	06/07/07	Oyster	ST-676	cluster_12	105	5049334	799420	401329	144689	5	10	0
MIRO01	GCSL_R75	USA, VA	23/08/07	Oyster	ST-676	cluster_12	87	5014250	799091	313333	142591	5	11	0
MIRP01	GCSL_R76	USA, VA	23/08/07	Oyster	ST-676	cluster_12	82	5017829	799375	313262	114659	5	11	0
MIRQ01	GCSL_R77	USA, VA	23/08/07	Oyster	ST-676	cluster_12	76	5014463	799420	443710	142549	5	10	0
SRR1118664	CDC_K4638	USA, NY	25/09/06	Stool	ST-676	cluster_12	93	5027622	799272	443765	189535	5	9	2.51
SRR1118665	CDC_K4588	USA, ME	26/07/06	Stool	ST-676	cluster_12	58	5017497	801676	443696	189175	5	9	4.63
LCVL01	VH3	Greece	2007	Environmental	ST--	cluster_120	67	4955051	592284	370587	124464	6	12	0
LFWI01	M-17-6	Canada	2015	Mollusk	ST-1346	cluster_121	70	5245836	673671	458622	299211	5	9	0
JNUN02	CFSAN007459	USA, MD	2010	Oyster	ST-768	cluster_122	45	5205568	1317831	425650	265901	4	8	0
MIQQ01	GCSL_R17	USA, FL	30/04/07	Oyster	ST-536	cluster_123	100	5010966	653013	400267	179823	5	9	0.04
AWLE01	S060	China	1992	Clinical	ST--	cluster_124	734	4936722	99726	13928	7304	107	231	0
AWJX01	S098	USA	1997	Seafood	ST--	cluster_125	659	5297296	107368	17405	9101	96	201	0
MITL01	CDC_K4981	USA, OK	12/03/07	Other	ST-748	cluster_126	135	4928549	399573	177698	85166	9	19	0
AWHO01	S165	China	2007	Environmental	ST-1010	cluster_127	608	5025645	83969	22119	12053	70	147	0
AWIN01	S139	China	2006	Seafood	ST-154	cluster_129	579	5014838	67972	22603	11504	74	152	0
MIQN01	GCSL_R12	USA, LA	27/03/07	Oyster	ST-32	cluster_13	116	5051895	806832	321940	133866	5	11	0
MIQS01	GCSL_R26	USA, NJ	16/06/07	Oyster	ST-32	cluster_13	134	5067308	819245	313874	133254	5	12	0
MIRA01	GCSL_R47	USA, AL	20/04/07	Oyster	ST-32	cluster_13	150	5064253	617921	313873	133283	6	12	0
MIUK01	CDC_K5330	USA, TX	23/04/07	ND	ST--	cluster_130	142	5168043	662056	192823	109362	8	17	0
JMMQ01	SG176	USA, GA	2006	Water	ST--	cluster_131	48	4952407	1227949	394204	235335	4	8	0.06
AWIK01	S142	China	2006	Seafood	ST-1008	cluster_132	594	5174666	85562	21560	11699	78	157	0
JNSU01	CFSAN007437	USA, MD	31/05/12	Stool	ST--	cluster_133	243	5051373	191286	59799	34514	28	54	0
JALI01	VP2007-007	USA	2007	Water	ST-307	cluster_134	695	5041389	71198	13214	7273	121	246	0
MIRN01	GCSL_R74	USA, VA	23/08/07	Oyster	ST-108	cluster_135	100	5041370	917285	521828	179493	4	9	0

ID	Alias	Geographical origin	Date	Source	ST	Cluster	No. Contigs	Total length	Largest contig	N50	N75	L50	L75	No. Ns/100 kbp
JALL01	M0605	Mexico, Sinaloa	22/07/13	Shrimp	ST-539	cluster_136	116	5429407	387292	121988	56979	12	29	14.91
JNSV01	CFSAN007438	USA, MD	08/06/12	Wound	ST--	cluster_137	212	5009364	135631	57425	29671	29	59	0
LFWJ01	S195-7	Canada	2007	Seafood	ST-1187	cluster_138	83	5224385	729533	324181	160769	6	12	0
AWJG01	S119	China	1999	Clinical	ST-224	cluster_139	718	5197930	65902	13018	6963	116	251	0
AOPD01	3644	USA, WA	2007	Stool	ST--	cluster_14	411	5028535	100260	31249	16843	53	108	45.7
AOPI01	EN9701072	USA, WA	1997	Stool	ST-43	cluster_14	425	4966234	95620	27006	14502	58	118	48.63
AWJV01	S101	USA	1990	Seafood	ST-43	cluster_14	595	5011175	88616	19913	10681	80	165	0
JNTC01	CFSAN007445	USA, MD	12/06/12	Stool	ST--	cluster_14	203	4985143	240943	56456	33395	26	54	0
JYJT01	10-4255	Canada, BC	2006	Clinical	ST-43	cluster_14	197	5091364	525731	239950	131582	7	13	0
JZAQ01	09-3217	Canada, BC	2009	Clinical	ST-43	cluster_14	155	5059362	553445	319036	105955	6	13	0
LHBF01	CFSAN018763	USA	2004	Missing	ST-43	cluster_14	81	5045122	438113	216660	112505	8	15	1.51
LOBT01	A4EZ700	Canada, BC	2004	Clinical	ST-43	cluster_14	76	5127080	536301	319036	105733	6	13	0
LOHO01	A4EZ724	Canada, BC	2004	Clinical	ST-43	cluster_14	72	5058285	525916	292301	112723	7	13	0.02
LPVN01	C148	Canada, BC	2008	Clinical	ST-43	cluster_14	80	5065366	437726	146049	88627	10	21	0
LQCS01	A5Z860	Canada, BC	2005	Clinical	ST-43	cluster_14	68	5094605	577342	319006	112723	6	12	0
LRAI01	05-3133	Canada, AB	2005	Clinical	ST-43	cluster_14	64	5022875	525916	292212	112723	7	13	0
LRFP01	A2EZ614	Canada, BC	2002	Clinical	ST-43	cluster_14	67	5065489	525750	254300	112723	7	14	0
LRSU01	F1419	Canada, BC	2006	Clinical	ST-43	cluster_14	64	5031127	525770	244430	112723	7	14	0
LRTC01	A3EZ710	Canada, BC	2003	Clinical	ST-43	cluster_14	77	5122387	533835	319006	106263	6	13	0
LRTD01	A3EZ711	Canada, BC	2003	Clinical	ST-43	cluster_14	72	5127994	533835	319035	112723	6	13	0
LRTF01	A3EZ799	Canada, BC	2003	Clinical	ST-43	cluster_14	73	5044575	525750	292206	112723	7	13	0
LRTI01	A1EZ952	Canada, BC	2001	Clinical	ST-43	cluster_14	65	5066903	525916	254552	112723	7	14	0
MIVB01	CDC_K5579	USA, IN	ND	Stool	ST-43	cluster_14	169	5072634	576884	191195	79222	8	18	0
MISS01	CDC_K4558G	USA, LA	28/08/06	Wound	ST-1143	cluster_140	120	5032007	554062	314740	158365	6	12	0
MBTR01	KVp10	Sweden	2007	Environmental	ST--	cluster_141	57	5008534	521527	237565	105586	8	16	0
MISQ01	CDC_K4556R	USA, LA	23/10/06	Wound	ST-744	cluster_142	135	5149094	820212	190507	108066	8	16	0
AWLP01	S046	Spain	1982	Environmental	ST--	cluster_143	981	5233467	57337	11177	6317	139	295	0
AXNP01	VIP4-0443	Hong Kong	2008	Big.eye.fish	ST--	cluster_144	1156	4872707	37085	7337	3888	205	427	0.47
JPLV01	FIM-S1708+	Mexico, Hermosillo	20/01/14	Sediment	ST-1167	cluster_147	79	5246988	629794	174266	103056	10	19	0
JNTD01	CFSAN007446	USA, MD	05/08/12	Wound	ST-678	cluster_148	137	4975370	409634	107992	51431	15	31	0
LFXK01	M-14-5	Canada	2014	Mussel	ST--	cluster_149	93	5060270	435762	279336	178222	8	13	0
AWJI01	S117	Japan	1984	Clinical	ST-262	cluster_15	773	5064437	70982	12496	6955	114	247	0
AWLK01	S053	Thailand	1990	Clinical	ST-262	cluster_15	790	5005317	57343	13539	7463	110	233	0
AWLQ01	S045	Thailand	1990	Clinical	ST--	cluster_15	518	5042752	105459	21335	11619	76	157	0
AWLR01	S044	Thailand	1990	Clinical	ST-262	cluster_15	756	5046675	51241	13374	7455	117	244	0

ID	Alias	Geographical origin	Date	Source	ST	Cluster	No. Contigs	Total length	Largest contig	N50	N75	L50	L75	No. Ns/100 kbp
F3305-VP	F3305-VP	UK-Norfolk	2005	Clinical	ST-262	cluster_15	60	5100438	408891	194519	87342	10	18	0
LRFX01	W501	Canada, BC	2006	Clinical	ST-635	cluster_151	40	4963903	988687	509565	168432	4	8	0
AOCL01	PCV08-7	Malaysia, Selangor	2008	Seafood	ST-808	cluster_152	83	5184985	704302	262059	131525	7	13	0
AWIH01	S145	China	2006	Seafood	ST--	cluster_153	455	5169969	96336	23804	12680	64	138	0
JNTF01	CFSAN007448	USA, PA	03/08/12	Wound	ST-679	cluster_154	232	5294940	291397	61073	36356	27	54	0
AWLS01	S043	Thailand	ND	Clinical	ST-1017	cluster_155	725	5097605	72186	14730	7492	103	224	0
AWHT01	S160	China	2006	Seafood	ST-329	cluster_156	541	4784022	62467	17257	10726	81	169	0
JEMS01	VP49	India, Mangalore	2008	Seafood	ST--	cluster_157	137	5047822	198771	68277	35228	24	49	0
14-1499-VP	14-1499-VP	UK	18/08/14	Oyster	ST-1157	cluster_158	113	5121520	347717	122983	66518	13	26	0
PY233	PY233	Spain	2006	Environmental	ST-169	cluster_159	38	5254029	952729	450349	197320	4	8	0
AWMR01	S018	China	1993	Clinical	ST-120	cluster_16	729	5158932	77651	15209	8788	105	217	0
AWMT01	S016	China	1992	Clinical	ST-120	cluster_16	595	5105432	72336	15462	8875	101	206	0
LKQA01	CFSAN025053	Peru	2009	ND	ST-120	cluster_16	49	5126292	621462	313618	194033	6	12	0.92
LKQB01	281-09-CFSAN025052	Peru	2009	ND	ST-120	cluster_16	42	5129398	535557	280966	178389	7	13	0
LKQC01	CFSAN025054	Peru	2009	ND	ST-120	cluster_16	34	5179121	714305	453287	231801	5	9	0
LKQD01	CFSAN025055	Peru	2009	ND	ST-120	cluster_16	47	5123197	569747	404432	178400	5	11	1.13
LKQE01	CFSAN025056	Peru	2009	ND	ST-120	cluster_16	35	5130775	572247	391859	234755	6	10	0
LKQF01	CFSAN025057	Peru	2009	ND	ST-120	cluster_16	35	5136853	1331797	453287	179585	4	8	0
LKQG01	CFSAN025058	Peru	2009	ND	ST-120	cluster_16	35	5126956	690962	467870	234996	5	9	0
LKQH01	CFSAN025059	Peru	2009	ND	ST-120	cluster_16	40	5126321	926197	376159	178389	5	11	0
LKQI01	CFSAN025060	Peru	2009	ND	ST-120	cluster_16	52	5126855	695584	265563	178389	7	13	1.81
LKQJ01	CFSAN025061	Peru	2009	ND	ST-120	cluster_16	34	5129762	1238720	467872	235116	4	8	0.84
LKQK01	CFSAN025062	Peru	2009	ND	ST-120	cluster_16	35	5128953	777040	467870	260477	5	8	0
LKQL01	CFSAN025063	Peru	2009	ND	ST-120	cluster_16	35	5220707	850506	455995	257547	5	8	0
LKQM01	CFSAN025064	Peru	2009	ND	ST-120	cluster_16	40	5130119	850548	337873	179358	5	11	0
LKQN01	CFSAN025065	Peru	2009	ND	ST-120	cluster_16	44	5126976	690964	284637	193967	6	11	0
LKQO01	CFSAN025066	Peru	2009	ND	ST-120	cluster_16	39	5132315	572884	330290	200648	6	10	0
LKQP01	CFSAN025067	Peru	2009	ND	ST-120	cluster_16	45	5130983	621466	366735	193981	5	10	0
LKQQ01	CFSAN025068	Peru	2009	ND	ST-120	cluster_16	69	5125547	535965	221959	118781	7	14	0
LKQR01	P306-CFSAN029653	Peru	2009	Oysters	ST-120	cluster_16	112	5122821	244230	101266	54475	17	35	0
LKQS01	Guillen151-CFSAN029654	Peru	2009	ND	ST-120	cluster_16	104	5126587	330299	152415	67163	12	25	0

ID	Alias	Geographical origin	Date	Source	ST	Cluster	No. Contigs	Total length	Largest contig	N50	N75	L50	L75	No. Ns/100 kbp
LKQT01	P310-CFSAN029656	Peru	2009	Oysters	ST-120	cluster_16	80	5127031	344208	145779	88843	12	23	0
AWJZ01	S096	South Korea	1999	Clinical	ST-217	cluster_160	655	5172050	84598	17831	10792	87	180	0
MITJ01	CDC_K4858	USA, HI	15/09/06	Stool	ST-283	cluster_161	89	4961237	674964	322414	188337	5	10	0
AOPG01	901128	USA, WA	1997	Stool	ST--	cluster_162	403	5255365	114356	31795	18288	51	106	42.18
AWLW01	S039	China	ND	Clinical	ST-321	cluster_163	795	5025522	64946	13001	7139	122	248	0
AWLF01	S058	Japan	1970	Clinical	ST-143	cluster_164	499	5030729	97127	24420	12231	63	137	0
LHBJ01	CFSAN018767	USA	2004	Missing	ST-20	cluster_165	85	5085389	494861	185639	84180	9	21	0
JPLU01	FIM-S1392-	Mexico, Hermosillo	20/01/14	Sediment	ST-1183	cluster_166	14	5174919	2406161	1931617	1931617	2	2	157.05
SRR1118638	CDC_K4760	USA, VA	2006	Blood	ST--	cluster_167	72	5070185	692789	394007	197447	5	9	2.92
MISD01	GCSL_R126	USA, FL	14/10/07	Oyster	ST-1146	cluster_168	115	5138446	638551	244992	121910	7	14	0
AWHQ01	S163	Malaysia	2007	Seafood	ST-994	cluster_169	510	5170229	92400	23895	11960	71	146	0
AXNQ01	VIP4-0219	HongKong	2006	Seafood	ST-937	cluster_17	79	5178152	685414	211270	140424	7	14	0.1
JNTJ02	CFSAN007452	USA, MD	17/06/10	Stool	ST-8	cluster_17	37	5127281	1521871	527441	226104	3	7	0
JNTK02	CFSAN007453	USA, MD	07/07/10	Stool	ST-8	cluster_17	46	5124758	1265931	367090	179144	4	9	0
JNUG02	CFSAN012491	USA, MD	2010	Oyster	ST-8	cluster_17	35	5126325	995851	526001	233570	4	7	0.41
JNUH02	CFSAN012492	USA, MD	2010	Oyster	ST-8	cluster_17	34	5128956	1192043	485136	321434	4	7	0.62
JNUI02	CFSAN012493	USA, MD	2010	Oyster	ST-8	cluster_17	47	5123258	635564	329201	188242	6	11	0
JNUJ02	CFSAN012494	USA, MD	2010	Oyster	ST-8	cluster_17	34	5127159	1521578	527658	225576	3	7	0
MISR01	CDC_K4557	USA, LA	19/09/06	Stool	ST-799	cluster_17	94	5074019	652712	361205	185517	5	11	0
AWIE01	S148	China	2006	Seafood	ST--	cluster_170	641	5020168	68274	16810	8505	97	205	0
AWML01	S024	China	1998	Clinical	ST-566	cluster_171	422	5083890	77966	22981	13158	67	138	0
JMMT01	22702	USA, GA	1998	Sediment	ST--	cluster_172	43	4955222	786063	454662	179586	5	10	0
LHBI01	CFSAN018766	USA	2004	Missing	ST-9	cluster_173	60	5043748	686903	348215	139502	6	12	0
AWIM01	S140	China	2006	Seafood	ST--	cluster_174	549	5127974	96260	27809	12997	61	128	0
LRSY01	A0EZ383	Canada, BC	2000	Clinical	ST-638	cluster_175	37	5158965	804714	475179	183638	5	9	0
LRFT01	A5Z1022	Canada, BC	2005	Clinical	ST-15	cluster_176	35	5128457	847668	443357	224432	5	8	0
LFWP01	S349-10	Canada	2010	Seafood	ST-1516	cluster_177	74	5225795	953466	273670	171662	5	11	0
AWLL01	S052	Spain	1975	Seafood	ST--	cluster_178	591	4995322	94316	18199	9456	81	175	0
LIRS01	HS-06-05	Canada	2014	Clam	ST-614	cluster_179	162	5845987	915959	174007	48022	7	24	0
ACFN01	AQ4037	Maldives	1985	Clinical	ST--	cluster_18	164	4939804	241746	67710	37290	18	44	0
AWKA01	S095	China	1996	Clinical	ST-91	cluster_18	452	5074062	107387	25401	13733	64	133	0
AWMW01	S013	China	1996	Clinical	ST-91	cluster_18	462	5018967	144217	31330	17335	52	105	0
AWMX01	S012	Thailand	1990	Clinical	ST--	cluster_18	533	5075464	64363	18090	9970	86	178	0
AWMY01	S011	Thailand	1990	Clinical	ST--	cluster_18	371	5070047	135966	30146	16863	51	108	0

ID	Alias	Geographical origin	Date	Source	ST	Cluster	No. Contigs	Total length	Largest contig	N50	N75	L50	L75	No. Ns/100 kbp
AWMZ01	S009	Thailand	1987	Clinical	ST-91	cluster_18	509	5073685	75353	19460	10960	80	163	0
AWNA01	S008	Thailand	1987	Clinical	ST-91	cluster_18	440	5072709	91236	24941	13923	65	130	0
AWNB01	S005	Thailand	ND	Clinical	ST-91	cluster_18	416	5011836	113051	28304	14973	58	118	0
AWNC01	S004	Maldives	1985	Clinical	ST-96	cluster_18	704	5010468	41702	14023	7610	117	237	0
AWND01	S003	China	ND	Clinical	ST-91	cluster_18	465	5002256	89393	21622	13073	68	142	0
MIQX01	GCSL_R33	USA, LA	30/05/07	Oyster	ST-28	cluster_180	94	5043795	652624	350943	179583	5	10	0
LFWN01	S456-5	Canada	2012	Seafood	ST--	cluster_181	272	6099228	1726234	735358	104794	3	9	0
JNTL01	CFSAN007455	USA, MD	19/07/13	Stool	ST-896	cluster_182	217	5061021	251879	55165	34978	28	57	0
AWHP01	S164	USA	2007	Seafood	ST--	cluster_183	541	4985943	58506	20188	11474	80	160	0
JOKE01	13-028/A3	Vietnam	2013	Water	ST--	cluster_184	290	5388844	221100	103448	60491	17	34	0
AWHJ01	S171	China	2007	Environmental	ST-288	cluster_185	342	5146548	126842	35949	20454	46	92	0
AWIB01	S152	China	2006	Seafood	ST-547	cluster_186	588	5009076	54308	15084	8679	97	207	0
AWIL01	S141	China	2006	Seafood	ST--	cluster_187	505	5104762	78987	25799	13602	64	128	0
MCFR01	R10B2_71	USA, WA	1997	Oyster	ST--	cluster_188	550	5270223	372720	131833	95569	11	22	0
MISK01	GCSL_R143	USA, FL	01/11/07	Oyster	ST-743	cluster_189	148	4947784	864380	314716	100967	5	12	0
MIUH01	CDC_K5324G	USA, VA	17/06/07	Stool	ST-1132	cluster_19	294	5272305	404382	130569	61198	11	25	0
MIUI01	CDC_K5324W	USA, VA	17/06/07	Stool	ST-1132	cluster_19	174	5060135	345161	139910	74368	12	23	0
SRR1118612	CDC_K5324G	USA, VA	17/06/07	Stool	ST-1132	cluster_19	165	5084929	327721	119756	72394	14	27	3.47
JNUK02	CFSAN007456	USA, MD	2010	Oyster	ST-810	cluster_190	56	5206921	878464	480822	315414	4	8	0
MIRR01	GCSL_R86	USA, FL	13/08/07	Oyster	ST-737	cluster_191	116	4894547	380580	192233	119425	8	15	0
MIVG01	CDC_K5635	USA, MD	03/09/07	Wound	ST-1145	cluster_192	110	5070117	959187	517784	179298	4	9	0
AWIY01	S128	India	1999	Clinical	ST--	cluster_193	384	4949196	89271	30162	16073	56	110	0
JMMP01	K1275	USA, TX	2004	Blood	ST--	cluster_194	63	5114306	804279	301883	154976	5	12	0.02
LFYM01	ISF-29-3	Canada	2011	Shrimp	ST-1518	cluster_195	94	5368163	643369	255897	157670	6	12	0
LIRU01	S176-10	Canada		Seafood	ST--	cluster_196	82	5976345	653807	366469	152118	6	14	0
JPIP01	VPA-67	India, Andhra Pradesh	13/11/13	Water	ST--	cluster_197	1940	5007772	69723	3582	2050	422	882	0
BAVH01	TUMSAT_D06_S3	Thailand	ND	Shrimp	ST-413	cluster_198	69	5233673	1758462	560774	229120	3	6	26.9
AWLO01	S047	Spain	1981	Environmental	ST--	cluster_199	612	5084123	88327	24746	11804	67	142	0
AFBW01	10329	USA, WA	1998	Clinical	ST-36	cluster_2	33	5093003	1325518	730973	449852	3	5	0
AOOU01	97-10290	USA, WA	1997	Stool	ST-36	cluster_2	61	5103858	480672	236714	111874	7	14	0.12
AOOX01	846	USA, WA	2007	Oyster	ST-36	cluster_2	115	5084575	474131	264478	111285	7	14	0.08
AOPA01	3324	USA, WA	2007	Stool	ST-36	cluster_2	88	5098399	522532	348180	168497	6	12	0.16
AOPF01	12315	USA, WA	2006	Stool	ST-36	cluster_2	110	5090858	496297	270580	171304	7	12	0.04
AOPH01	EN2910	USA, WA	2000	Stool	ST-36	cluster_2	49	5092882	690250	398882	234567	5	10	0
AOPK01	EN9701173	USA, WA	1997	Stool	ST-36	cluster_2	54	5093059	871209	440310	171340	4	9	0

ID	Alias	Geographical origin	Date	Source	ST	Cluster	No. Contigs	Total length	Largest contig	N50	N75	L50	L75	No. Ns/100 kbp
AOPL01	EN9901310	USA, WA	1999	Stool	ST-36	cluster_2	61	5092164	871204	439695	163908	5	10	0
AWLX01	S038	USA	1982	Environmental	ST-59	cluster_2	840	5115149	53646	11986	7079	137	273	0
AWLY01	S037	USA	1994	Clinical	ST--	cluster_2	797	5062128	86662	12391	7157	125	261	0
AYSP01	10296	USA	1997	Stool	ST-36	cluster_2	198	5101141	198251	58269	32454	29	59	0
AYXP01	12310	USA, WA	2006	Stool	ST-36	cluster_2	103	5133901	432155	200999	80665	8	18	0
AZGS01	3256	USA	2007	Stool	ST-36	cluster_2	98	5085680	446881	269910	95429	8	17	0
G25	G25	Spain, Galicia	2012	Clinic	ST-36	cluster_2	44	5186540	1187033	419551	231895	4	8	0.46
G30	G30	Spain, Galicia	2012	Clinic	ST-36	cluster_2	42	5187399	1101532	581301	315147	3	6	0
G31	G31	Spain, Galicia	2012	Clinic	ST-36	cluster_2	55	5191332	1186717	458794	169976	4	9	0
G35	G35	Spain, Galicia	2012	Clinic	ST-36	cluster_2	53	5185751	1185104	335714	190331	5	10	0
G36	G36	Spain, Galicia	2012	Clinic	ST-36	cluster_2	44	5188301	1186627	561284	465525	4	6	0
G37	G37	Spain, Galicia	2012	Clinic	ST-36	cluster_2	54	5183508	676075	458751	180707	5	9	0
JAIJ01	SBR10290	USA	1997	Stool	ST-36	cluster_2	166	5113040	372887	66962	36056	22	48	0
JMMO01	K1461	USA, MA	2004	Stool	ST-36	cluster_2	65	5177972	589658	303097	127756	6	14	0.35
JNTM01	CFSAN006129	USA, MD	03/08/12	Stool	ST-36	cluster_2	284	4991166	229727	43911	25499	34	71	0
JNTN01	CFSAN006131	USA, MD	30/06/13	Stool	ST-36	cluster_2	281	4993343	132532	40074	19423	41	86	0
JNTO01	CFSAN006132	USA, MD	17/06/13	Stool	ST--	cluster_2	283	4975382	135180	35662	20527	43	89	0
JNTP01	CFSAN006133	USA, MD	05/07/13	Stool	ST-36	cluster_2	276	4981396	153424	42084	25099	36	74	0
JNTQ01	CFSAN006134	USA, MD	16/07/13	Stool	ST--	cluster_2	215	5065048	245587	50327	28815	30	62	0
JNTR01	CFSAN006135	USA, MD	21/07/13	Stool	ST--	cluster_2	250	4994309	161283	49481	24531	31	68	0
JNTS01	CFSAN007460	USA, MD	07/08/13	Stool	ST--	cluster_2	279	4997118	144966	39032	20328	44	88	0
JNTT01	CFSAN007461	USA, MD	27/08/13	Stool	ST-36	cluster_2	185	5037057	281548	79912	45229	20	41	0
JNTU01	CFSAN007462	USA, MD	02/06/13	Stool	ST--	cluster_2	558	4868147	70579	16268	8401	85	186	0
JNTV01	CFSAN006130	USA, MD	02/06/13	Stool	ST--	cluster_2	269	4987136	124245	39128	23351	40	80	0
JNTW01	CFSAN001611	USA, OR	1997	Environmental	ST-36	cluster_2	120	5140604	482159	134517	70658	10	24	0
JNTX01	CFSAN001612	USA, WA	1990	Clinical	ST-36	cluster_2	111	5068718	401003	117873	63590	13	27	0
JNTY01	CFSAN001614	USA, AK	2004	ND	ST-59	cluster_2	130	5207722	417409	135651	55101	12	26	0
JNUA01	CFSAN001618	USA, WA	1990	Clinical	ST-36	cluster_2	105	5076977	360900	153208	67367	12	24	0
JNUB01	CFSAN001619	USA, WA	1988	Environmental	ST-36	cluster_2	104	5079055	442770	136757	77171	12	24	0
JNUC01	CFSAN001620	USA, NY	1998	Environmental	ST-36	cluster_2	125	5061001	388055	112344	69665	12	27	0
JNUD01	K1203	USA, AK	2004	ND	ST-59	cluster_2	206	5178453	346740	97212	45126	17	36	0
JNUF01	CFSAN001613	USA, WA	1997	Clinical	ST-36	cluster_2	151	5026149	290532	100912	51249	15	32	0
JWSS01	10329	USA, WA	1998	Stool	ST-36	cluster_2	2	5149046	3316038	3316038	1833008	1	2	0
JWSV01	K1198	USA, AK	2004	Environmental	ST-59	cluster_2	8	5318060	2468441	972370	911315	2	3	0
JXUX01	10-7197	Canada, BC	2008	Clinical	ST-36	cluster_2	58	5091379	1072038	475405	376267	4	7	0
JXUY01	10-4303	Canada, BC	2000	Clinical	ST-36	cluster_2	53	5106706	761861	437054	404521	5	8	0

ID	Alias	Geographical origin	Date	Source	ST	Cluster	No. Contigs	Total length	Largest contig	N50	N75	L50	L75	No. Ns/100 kbp
JXUZ01	10-4298	Canada, BC	2001	Clinical	ST-36	cluster_2	77	5233482	998036	426178	161560	5	9	0
JXVA01	10-4293	Canada, BC	2002	Clinical	ST-36	cluster_2	59	5202137	681077	444499	315701	5	8	0
JXVB01	10-4288	Canada, BC	2003	Clinical	ST-36	cluster_2	62	5109495	1072264	548500	376400	4	7	0
JXVC01	10-4274	Canada, BC	2005	Clinical	ST-36	cluster_2	96	5115101	1072077	548500	248114	4	7	0
JXVD01	10-4248	Canada, BC	2006	Clinical	ST-36	cluster_2	117	5112922	1072174	548500	315446	4	7	0
JXVE01	10-4247	Canada, BC	2006	Clinical	ST-36	cluster_2	85	5124152	1083362	462063	232219	4	8	0
JXVF01	10-4246	Canada, BC	2006	Clinical	ST-36	cluster_2	76	5098304	648842	423840	376936	5	8	0
JXVG01	10-4245	Canada, BC	2006	Clinical	ST-36	cluster_2	72	5096818	648456	423844	232219	5	9	0
JXVH01	10-4242	Canada, BC	2006	Clinical	ST-36	cluster_2	75	5126721	752496	403279	117811	6	11	0
JXVI01	10-4241	Canada, BC	2006	Clinical	ST-36	cluster_2	58	5104300	1080786	548500	403489	4	7	0
JXVJ01	09-3216	Canada, BC	2009	Clinical	ST-36	cluster_2	79	5099993	941609	548674	376802	4	7	0
JXVK01	04-1290	Canada, AB	2004	Clinical	ST-36	cluster_2	98	5143276	1072183	548500	376238	4	7	0
LBHD01	MAVP-26	USA, MA	2013	Clinical	ST-36	cluster_2	30	5112994	1268488	809118	548782	3	5	2.15
LBHE01	MAVP-36	USA, MA	2013	Clinical	ST-36	cluster_2	32	5193870	1191635	809400	550114	3	5	1.35
LBHN01	MAVP-45	USA, MA	2013	Clinical	ST-36	cluster_2	24	5113194	1329274	812214	561936	3	4	13.89
LBHO01	MAVP-V	USA, MA	2011	Clinical	ST-36	cluster_2	39	5204156	1191363	764045	385855	3	6	12.27
LNTX01	A1EZ919	Canada, BC	2001	Clinical	ST-36	cluster_2	31	5094419	1072438	462093	376127	4	7	0
LPVB01	C143	Canada, BC	2008	Clinical	ST-36	cluster_2	30	5078280	1072089	548226	376534	4	7	0
LPVC01	C144	Canada, BC	2008	Clinical	ST-36	cluster_2	34	5078762	1072353	548500	315567	4	7	0
LPVM01	C147	Canada, BC	2008	Clinical	ST-36	cluster_2	36	5078771	1072353	521092	376132	4	7	0
LQCE01	A5Z652	Canada, BC	2005	Clinical	ST-36	cluster_2	32	5082632	1095187	556943	315570	3	6	0
LQCT01	A5Z878	Canada, BC	2005	Clinical	ST-36	cluster_2	36	5079776	998154	548591	376132	4	7	0
LQCU01	A5Z905	Canada, BC	2005	Clinical	ST-36	cluster_2	31	5226793	736492	570484	261316	4	8	0
LQCV01	A5Z924	Canada, BC	2005	Clinical	ST-36	cluster_2	38	5076261	969419	376542	170704	5	10	0
LRFM01	A0EZ608	Canada, BC	2000	Clinical	ST-36	cluster_2	28	5096832	1071920	548505	376796	4	7	0
LRFQ01	A2EZ715	Canada, BC	2002	Clinical	ST-36	cluster_2	31	5103078	1072221	548679	376126	4	7	0
LRFU01	F4395	Canada, BC	2006	Clinical	ST-36	cluster_2	34	5134947	1072089	548500	315573	4	7	0
LRFY01	H11523	Canada, BC	2006	Clinical	ST-36	cluster_2	34	5077113	1072160	548500	376139	4	7	0
LRFZ01	H64024	Canada, BC	2006	Clinical	ST-36	cluster_2	33	5080527	1072353	548500	232219	4	7	0
LRGA01	T8994	Canada, BC	2006	Clinical	ST-36	cluster_2	34	5076710	1094973	548500	315433	3	6	0
LRJZ01	M59787	Canada, BC	2006	Clinical	ST-36	cluster_2	34	5076618	1072264	462077	229078	4	8	0
LRST01	H18983	Canada, BC	2006	Clinical	ST-36	cluster_2	48	5085869	1072347	548500	187447	4	8	0
LRSW01	09-3219	Canada, BC	2009	Clinical	ST-36	cluster_2	38	5074950	1072436	462063	232219	4	8	0
LRSZ01	A1EZ679	Canada, BC	2001	Clinical	ST-36	cluster_2	31	5110204	1072438	548505	376126	4	7	0
LRTA01	A2EZ523	Canada, BC	2002	Clinical	ST-36	cluster_2	33	5093534	1072438	462105	315561	4	7	0
MISZ01	CDC_K4639G	USA, NY	16/10/06	Stool	ST-36	cluster_2	122	5092291	858270	548356	179304	4	9	0

ID	Alias	Geographical origin	Date	Source	ST	Cluster	No. Contigs	Total length	Largest contig	N50	N75	L50	L75	No. Ns/100 kbp
MITA01	CDC_K4639W	USA, NY	16/10/06	Stool	ST-36	cluster_2	114	5078734	858270	548241	179468	4	9	0
MITY01	CDC_K5278	USA, WA	25/06/07	Stool	ST-36	cluster_2	112	5067491	858277	548384	179304	4	9	0
MIUA01	CDC_K5280	USA, WA	11/07/07	Stool	ST-36	cluster_2	110	5083288	866708	536709	179333	4	9	0
MIUB01	CDC_K5281	USA, WA	13/07/07	Stool	ST-36	cluster_2	115	5077351	866703	548238	179448	4	9	0
MIUE01	CDC_K5308	USA, AK	14/05/07	Stool	ST-36	cluster_2	142	5095192	781430	315048	152425	5	12	0
MIUJ01	CDC_K5328	USA, IN	ND	Stool	ST-36	cluster_2	118	5090169	869543	322231	153978	5	10	0
MIUM01	CDC_K5345G	USA, IA	07/08/07	Stool	ST-36	cluster_2	112	5148490	858270	548356	179304	4	9	0
MIUN01	CDC_K5345W	USA, IA	07/08/07	Stool	ST-36	cluster_2	105	5125535	858180	548245	191923	4	8	0
MIUO01	CDC_K5346	USA, PA	21/08/07	ND	ST-36	cluster_2	126	5087977	858269	548384	179304	4	9	0
MIUQ01	CDC_K5429	USA, NV	09/08/07	Stool	ST-36	cluster_2	118	5079150	871317	536614	179337	4	9	0
MIUR01	CDC_K5433	USA, WA	24/07/07	Stool	ST-36	cluster_2	118	5073057	858270	525700	179448	4	9	0
MIUT01	CDC_K5437	USA, WA	02/09/07	Stool	ST-36	cluster_2	126	5068632	858270	548245	179333	4	9	0
MIUW01	CDC_K5456	USA, WA	ND	Stool	ST-36	cluster_2	114	5080809	858270	548195	179451	4	9	0
MIUX01	CDC_K5457	USA, WA	07/08/07	Stool	ST-36	cluster_2	97	5075024	781423	536921	179439	4	9	0
MIUZ01	CDC_K5512	USA, OK	14/06/07	Stool	ST-36	cluster_2	122	5082991	781423	322224	161150	5	10	0
MIVF01	CDC_K5629	USA, GA	18/11/07	Stool	ST-36	cluster_2	119	5077945	858270	548240	179429	4	9	0
MIVH01	CDC_K5638	USA, MD	ND	Stool	ST-36	cluster_2	125	5144324	858270	548356	179452	4	9	0
MIUP01	CDC_K5428	USA, NV	06/07/07	Stool	ST-199	cluster_20	139	5092714	546503	312872	156220	7	12	0
PY194	PY194	Spain	2007	Environmental	ST-199	cluster_20	57	5046957	551104	313864	155494	6	11	0
AWIF01	S147	China	2006	Seafood	ST--	cluster_200	655	5120789	71186	17214	9201	93	195	0
JDFN01	VPTS-2010	USA	2010	Water	ST-6	cluster_201	659	5218338	74713	14217	7740	107	231	0
JDFO01	VPTS-2010_2	USA	2010	Water	ST--	cluster_202	713	4992918	66819	12510	7169	118	249	0
AWJK01	S115	Thailand	1991	Clinical	ST--	cluster_203	854	5144095	69430	11577	6262	131	279	0
MIQO01	GCSL_R13	USA, LA	27/03/07	Oyster	ST-732	cluster_204	161	5122134	619470	179238	97355	7	16	0
LASL01	09-4681	Canada, NB	2009	Clinical	ST-632	cluster_205	49	5177998	1740325	536325	248786	3	6	0
MIRH01	GCSL_R57	USA, WA	14/07/07	Oyster	ST-1148	cluster_206	101	5100768	588341	314409	205336	6	11	0
LFWG01	ISF-25-6	Canada	2010	Mollusk	ST--	cluster_207	71	5077802	1408096	408976	231989	4	7	0
LIRR01	ISF-54-12	Canada	2011	Imported shrimp	ST--	cluster_208	74	5041359	871376	335149	174202	5	10	0
AOPJ01	EN9701121	USA, WA	1997	Stool	ST--	cluster_21	378	5107306	126153	32971	18685	46	97	43.09
AWIX01	S129	Japan	1984	Clinical	ST-50	cluster_21	659	5040562	116955	23715	12370	59	131	0
AWJU01	S104	USA	1997	Clinical	ST-50	cluster_21	719	5174530	54282	14072	7516	111	235	0
JNTZ01	CFSAN001617	USA, WA	1997	Clinical	ST-50	cluster_21	126	5175345	494581	131980	82411	13	26	0
JYJU01	10-4287	Canada, BC	2003	Clinical	ST-50	cluster_21	333	5269035	877315	509297	255128	5	8	0
LHBB01	CFSAN001174	USA	2004	Environmental	ST-4	cluster_21	88	5148437	301275	122650	77194	14	27	0
LOSG01	NSV5736	USA	ND	ND	ST-50	cluster_21	3	5332288	2511061	1907361	1907361	2	2	0
LRFN01	A0EZ664	Canada, BC	2000	Clinical	ST-50	cluster_21	37	5151804	878804	561155	401414	4	7	0

ID	Alias	Geographical origin	Date	Source	ST	Cluster	No. Contigs	Total length	Largest contig	N50	N75	L50	L75	No. Ns/100 kbp
LRFO01	A0EZ713	Canada, BC	2000	Clinical	ST-50	cluster_21	42	5151966	878151	461183	255579	5	8	0
LRTB01	A3EZ634	Canada, BC	2003	Clinical	ST-50	cluster_21	41	5147837	725547	461307	255094	5	8	0
LRTE01	A3EZ770	Canada, BC	2003	Clinical	ST-50	cluster_21	47	5177526	876648	542663	396771	5	7	0
MIUY01	CDC_K5485	USA, NC	08/07/07	Other	ST-50	cluster_21	128	5151277	664216	321792	153569	5	11	0
AXNR01	VIP4-0444	Hong Kong	2008	Big.eye.fish	ST--	cluster_210	106	5271920	895266	180240	78603	8	20	0.02
MITV01	CDC_K5126	USA, MS	21/05/07	Stool	ST-1131	cluster_211	152	5143760	659285	261043	82204	6	15	0
JDFL01	VPCR-2009	USA	2009	Water	ST--	cluster_212	191	5090393	404859	92465	61049	16	33	0
JNUL02	CFSAN007457	USA, MD	2010	Oyster	ST-811	cluster_213	47	5183716	750802	417837	166826	5	10	0
MISC01	GCSL_R125	USA, FL	14/10/07	Oyster	ST-739	cluster_214	150	5078459	566894	163207	71668	10	21	0
LQCB01	04-2192	Canada, Saskatchewan	2004	Clinical	ST-629	cluster_215	35	5245730	1024898	464267	316019	4	7	0
JNTE01	CFSAN007447	USA, MD	10/08/12	Ear	ST-162	cluster_216	214	4925201	308217	52439	28872	26	55	0
LBHG01	MAVP-M	USA, MA	2011	Clinical	ST-1127	cluster_217	287	5256857	911708	402360	169341	5	10	3.07
JMMR01	J-C2-34	USA, NC	1998	Sediment	ST--	cluster_218	91	5150449	749992	268853	121787	6	14	0.17
AWHM01	S167	China	2007	Environmental	ST-490	cluster_219	484	5160988	86253	21981	11415	70	152	0
MISH01	GCSL_R136	USA, SC	21/11/07	Oyster	ST-775	cluster_22	139	5098402	537286	168542	72887	10	22	0
MISI01	GCSL_R137	USA, SC	21/11/07	Oyster	ST-775	cluster_22	127	5107378	537509	141391	72369	11	23	0
MISJ01	GCSL_R138	USA, SC	21/11/07	Oyster	ST-775	cluster_22	148	5108840	537343	160436	74322	10	21	0
MITS01	CDC_K5067	USA, SD	28/04/07	Stool	ST-775	cluster_22	143	5028582	507354	150907	72887	10	22	0
SRR1118651	GCSL_R136	USA, SC	21/11/07	Oyster	ST-775	cluster_22	125	5108648	436773	160505	74398	10	21	4.02
AWJA01	S125	USA	1997	Seafood	ST-131	cluster_23	397	5086946	94210	27219	15592	58	119	0
AZIQ01	970107	USA	1997	Water	ST-131	cluster_23	215	5099912	169479	64091	37108	26	50	0
AONA01	49	USA, WA	2007	Oyster	ST--	cluster_24	363	5131690	129582	29474	16509	53	111	56.57
LFWO01	M-13-3	Canada	2015	Mollusk	ST-137	cluster_24	58	5233576	918818	427514	237368	4	8	0
090-96_1996_Peru	090-96-70	Peru	1996	Clinical	ST-265	cluster_25	35	5096451	1193063	539238	244618	4	7	0
AWME01	S031	Japan	1984	Clinical	ST-189	cluster_25	360	4993525	124465	35058	19750	47	92	0
BB2OPP.fna	BB22OP	Bangladesh	1980s	Environmental	ST-88	cluster_25	2	5103524	3297305	3297305	1806219	1	2	0
C18_2007_Peru	C18	Peru	2007	Clinical	ST-265	cluster_25	48	5021096	1193219	401280	159867	4	8	0
C18-245-07	C18-245	Peru	2007	Clinical	ST-265	cluster_25	48	5021096	1193219	401280	159867	4	8	0
C21-1262-07	C21-1262-07	Peru	2007	Clinical	ST-265	cluster_25	48	5023956	1193005	539211	244589	4	7	0
C21_2007_Peru	C21	Peru	2007	Clinical	ST-265	cluster_25	48	5023956	1193005	539211	244589	4	7	0
C4_1995_Peru	C4	Peru	1995	Clinical	ST-88	cluster_25	48	5114804	719534	412430	204240	5	9	0
C4-324-95	C4-324-95	Peru	1995	Clinical	ST-88	cluster_25	48	5114804	719534	412430	204240	5	9	0
C5_1995_Peru	C5	Peru	1995	Clinical	ST-88	cluster_25	43	5118365	719448	487186	176597	5	9	0
C5-326-95	C5-326-95	Peru	1995	Clinical	ST-88	cluster_25	43	5118365	719448	487186	176597	5	9	0
C6_1996_Peru	C6	Peru	1996	Clinical	ST-88	cluster_25	42	5118851	718872	412438	221256	5	9	0

ID	Alias	Geographical origin	Date	Source	ST	Cluster	No. Contigs	Total length	Largest contig	N50	N75	L50	L75	No. Ns/100 kbp
C6-267-96	C6-267-96	Peru	1996	Clinical	ST-88	cluster_25	42	5118851	718872	412438	221256	5	9	0
C7_1996_Peru	C7	Peru	1996	Clinical	ST-265	cluster_25	45	5098094	598579	499354	192689	5	9	0
C7-212-96	C7-212-96	Peru	1996	Clinical	ST-265	cluster_25	45	5098094	598579	499354	192689	5	9	0
C9-1257-07	C9-1257-07	Peru	2007	Clinical	ST-265	cluster_25	43	5026215	715933	539185	237068	5	8	0
C9_2007_Peru	C9	Peru	2007	Clinical	ST-265	cluster_25	43	5026215	715933	539185	237068	5	8	0
JFFP01	CFSAN001595	Peru	1996	Clinical	ST-265	cluster_25	116	5069445	358101	106956	79140	14	27	0
LFWF01	ISF-01-07	Canada	2010	Mollusk	ST-88	cluster_25	94	5052128	1081271	450650	164188	4	9	0
LPZT01	Gxw_9143	China, Guangxi	21/05/09	Stool	ST-265	cluster_25	57	5139256	949820	603826	226850	4	7	1.95
MIUL01	CDC_K5331	USA, GA	08/08/07	Stool	ST-265	cluster_25	95	5067258	871187	539086	168338	4	10	0
MIUV01	CDC_K5439	USA, WA	19/09/07	Stool	ST-189	cluster_25	106	5031538	871258	539098	182690	4	9	0
S030_India_1999.fsa	S030	India	1999	ND	ST-189	cluster_25	483	5092265	88285	24210	11812	69	140	0
Vp196	Vp196	Peru	2002	Environmental	ST-265	cluster_25	54	5994257	699266	499399	178871	6	11	0
Vp691-05	691-05	Peru	2005	Clinical	ST-265	cluster_25	59	5998819	1193419	414482	159801	5	11	0
AWLV01	S040	Thailand	1990	Clinical	ST-546	cluster_26	517	4986600	95118	19879	11080	71	153	0
JTGQ01	T12739	Canada, BC	2007	Clinical	ST-546	cluster_26	166	5037441	541626	179503	82982	9	19	0
AWHU01	S159	China	2006	Seafood	ST-1009	cluster_27	499	5041665	115328	24030	12730	69	143	0
AWHW01	S157	China	2006	Seafood	ST-1009	cluster_27	606	5037987	75611	17263	8889	93	195	0
G32	G32	Spain, Galicia	2012	Clinic	ST-1032	cluster_28	56	5007845	583932	192124	100940	8	16	1.38
PY350	PY350	Spain	2006	Environmental	ST-1032	cluster_28	48	5014270	890238	273630	157682	5	11	0
PY452	PY452	Spain	2007	Environmental	ST-1032	cluster_28	62	5010843	496496	178640	82342	9	19	0.34
PY456	PY456	Spain	2006	Environmental	ST-1032	cluster_28	49	5012260	666268	275369	157127	7	12	0.58
AWMU01	S015	China	1992	Clinical	ST--	cluster_29	545	5147850	74851	17831	10309	91	185	0
AWMV01	S014	China	1992	Clinical	ST--	cluster_29	545	5159245	65439	18650	10941	86	177	0
AOPB01	3355	USA, WA	2007	Stool	ST--	cluster_3	381	5119134	103336	29107	15610	56	114	49.28
AVPX01	NIHCB0757	Bangladesh	2006	Stool	ST-65	cluster_3	4	5262465	3378377	3378377	1870205	1	2	0
MITX01	CDC_K5277	USA, WA	ND	Stool	ST-65	cluster_3	109	5165700	615115	215075	104194	6	14	0
MITZ01	CDC_K5279	USA, WA	ND	Stool	ST-65	cluster_3	93	5172883	625873	245528	148062	6	13	0
MIUS01	CDC_K5435	USA, WA	11/08/07	Stool	ST-65	cluster_3	105	5168466	624781	215075	113415	6	14	0
MIUU01	CDC_K5438	USA, WA	09/09/07	Stool	ST-65	cluster_3	123	5154409	615149	313186	104262	6	13	0
MIVI01	CDC_K5701	USA, OR	09/09/07	Stool	ST-65	cluster_3	93	5157591	570619	315632	125815	6	12	0
SRR1118603	CDC_K5438	USA, WA	09/09/07	Stool	ST-65	cluster_3	88	5162750	570693	307872	118263	6	14	2.11
AWJF01	S120	Thailand	1990	Clinical	ST-327	cluster_30	782	5146827	61105	13137	7150	119	251	0
CFSAN045070_113477	CFSAN045070-113477	Spain, Galicia	10-2015	Clinic	ST-327	cluster_30	43	5179279	668543	269810	142412	6	13	0
JDFM01	VPTS-2009	USA	2009	Water	ST-1013	cluster_30	130	5074863	407313	167106	94385	10	20	0
N310	N310	Spain, Galicia	06/2016	Clinic	ST-327	cluster_30	50	5179327	1039396	247267	103767	6	15	0

ID	Alias	Geographical origin	Date	Source	ST	Cluster	No. Contigs	Total length	Largest contig	N50	N75	L50	L75	No. Ns/100 kbp
MIRS01	GCSL_R87	USA, FL	13/08/07	Oyster	ST-320	cluster_31	111	5180717	662109	408986	173721	5	11	0
MIRT01	GCSL_R88	USA, FL	13/08/07	Oyster	ST-320	cluster_31	90	5184181	841264	409230	161667	5	10	0
SRR1118662	CDC_K4760	ND	2006	Blood	ST-320	cluster_31	93	5191370	810359	409318	135637	5	10	3.38
AWJQ01	S109	China	1993	Clinical	ST-326	cluster_32	395	5051546	131681	38985	23802	40	79	0
AWMO01	S021	India	1999	Clinical	ST-326	cluster_32	551	5212928	86989	21666	12664	71	153	0
AWMP01	S020	China	1994	Clinical	ST--	cluster_32	421	5182867	111628	32890	16009	50	107	0
JZAN01	07-2965	Canada, AB	2007	Clinical	ST-326	cluster_32	85	5216789	943920	557313	365679	4	7	0
JNSY01	CFSAN007441	USA, MD	15/06/12	Wound	ST-113	cluster_33	286	4851619	135371	37940	23151	40	81	0
JNSZ01	CFSAN007442	USA, MD	10/07/12	Stool	ST-113	cluster_33	285	4852478	169072	42767	22702	37	76	0
JNTA01	CFSAN007443	USA, MD	23/07/12	Ear	ST-113	cluster_33	283	4856640	135371	40377	21731	38	79	0
MIRI01	GCSL_R59	USA, ME	23/07/07	Oyster	ST-113	cluster_33	128	4953641	516389	207497	90263	8	17	0
AWJR01	S108	Thailand	1990	Clinical	ST--	cluster_34	674	5075003	70551	15384	8062	105	218	0
AWJS01	S106	Thailand	1990	Clinical	ST--	cluster_34	609	5072077	56432	15464	8410	101	210	0
MIRM01	GCSL_R65	USA, ME	23/07/07	Oyster	ST-1150	cluster_35	111	5065957	864658	207247	117954	6	13	0
MISU01	CDC_K4588	USA, ME	26/07/06	Stool	ST-1150	cluster_35	118	5014336	864658	207438	117995	6	13	0
LHBC01	CFSAN018760	USA	2004	Missing	ST-61	cluster_36	61	4986443	561504	252511	124269	7	13	0
MIQZ01	GCSL_R45	USA, WA	2007	Oyster	ST-61	cluster_36	101	4973945	526512	253663	116524	7	14	0
AWJL01	S114	India	1998	Clinical	ST-83	cluster_37	698	5207427	63998	15194	8153	107	225	0
AWJM01	S113	India	1990	Clinical	ST-83	cluster_37	829	5136991	55379	15260	7676	102	216	0
AWJN01	S112	Japan	1951	Clinical	ST-1	cluster_37	683	5033228	59222	16061	8531	98	210	0
AWJO01	S111	Thailand	1990	Clinical	ST-83	cluster_37	667	5140602	82313	15938	8153	100	215	0
AWJP01	S110	India	1999	Clinical	ST-83	cluster_37	667	5117452	47015	14255	8066	111	230	0
BBQD01	NBRC12711	Japan	ND	Stool	ST-1	cluster_37	39	4991873	698502	381903	215279	5	10	0
LATW01	ATCC17802	Japan	1951	ND	ST-1	cluster_37	51	5067729	697590	366570	141395	5	11	0.45
AWIG01	S146	China	2006	Seafood	ST-12	cluster_38	527	5023056	80893	24916	12733	63	134	0
LHBD01	CFSAN018761	USA	2004	Missing	ST-12	cluster_38	37	5284763	836021	564826	231737	4	8	0
LHBE01	CFSAN018762	USA	2004	Missing	ST-12	cluster_38	505	5309702	91986	21464	11232	77	164	0.06
MIQR01	GCSL_R21	USA, TX	04/05/07	Oyster	ST-12	cluster_38	79	5123977	899189	477172	190435	4	8	0
MIRV01	GCSL_R96	Canada, PEI	31/07/07	Oyster	ST-1152	cluster_39	111	4931576	491394	246453	145899	8	14	0
MISB01	GCSL_R111	Canada, PEI	31/07/07	Oyster	ST-1152	cluster_39	95	4925222	867721	234627	115822	7	14	0
SRR1118656	CDC_K4857W	USA, HI	28/01/07	Stool	ST-1152	cluster_39	105	4940614	430829	234706	116839	8	15	1.97
AOPC01	3631	USA, WA	2007	Stool	ST--	cluster_4	447	5119359	110051	29816	16265	51	108	45.46
AOPE01	3646	USA, WA	2007	Stool	ST--	cluster_4	410	5110244	135298	34625	16888	46	99	48.59
JYJQ01	09-4663	Canada, BC	2009	Clinical	ST-417	cluster_4	148	5217021	660156	403392	186650	5	10	0
JYJV01	10-7205	Canada, BC	2008	Clinical	ST-417	cluster_4	146	5217627	625697	315571	179247	6	11	0
JZAP01	08-7626	Canada, AB	2008	Clinical	ST-417	cluster_4	121	5207542	699964	315699	179669	6	11	0

ID	Alias	Geographical origin	Date	Source	ST	Cluster	No. Contigs	Total length	Largest contig	N50	N75	L50	L75	No. Ns/100 kbp
LASH01	09-3218	Canada, BC	2009	Clinical	ST-417	cluster_4	89	5204568	625275	418558	186640	6	10	0
LASI01	09-4434	Canada, AB	2009	Clinical	ST-417	cluster_4	80	5194240	731692	425238	206186	5	9	0
LASJ01	09-4660	Canada, BC	2009	Clinical	ST-417	cluster_4	73	5191811	699958	315795	208946	6	11	0
LASK01	09-4664	Canada, BC	2009	Clinical	ST-417	cluster_4	89	5196673	661978	425466	206147	5	10	0
LNTR01	09-4661	Canada, BC	2009	Clinical	ST-417	cluster_4	93	5195946	731944	403281	206255	5	10	0
LPVA01	C142	Canada, BC	2008	Clinical	ST-417	cluster_4	52	5210898	467672	334240	196740	7	12	0
LPVK01	C145	Canada, BC	2008	Clinical	ST-417	cluster_4	47	5176962	910199	403418	206186	5	9	0
LPVU01	C150	Canada, BC	2008	Clinical	ST-417	cluster_4	53	5209864	1124884	334486	151629	5	12	0.02
LQCC01	09-4666	Canada, BC	2009	Clinical	ST-417	cluster_4	44	5172328	699958	425238	186642	5	10	0
LRFL01	09-4665	Canada, BC	2009	Clinical	ST-417	cluster_4	55	5169946	625399	307466	177762	6	12	0
LRSX01	09-1772	Canada, AB	2009	Clinical	ST-417	cluster_4	53	5173304	1146110	315980	179833	5	11	0
LRTH01	09-4662	Canada, BC	2009	Clinical	ST-417	cluster_4	47	5176120	840362	315437	187468	5	10	0
JNSW01	CFSAN007439	USA, DE	17/06/12	Stool	ST--	cluster_40	184	5031601	199490	66979	33741	26	52	0
JNSX01	CFSAN007440	USA, MD	11/07/13	Stool	ST-653	cluster_40	249	5013504	170480	39172	20394	38	82	0
MITT01	CDC_K5073	USA, MD	10/03/07	Stool	ST-750	cluster_40	117	5082152	600628	192573	100799	7	15	0
MIRC01	GCSL_R52	USA, WA	13/07/07	Oyster	ST-735	cluster_41	86	5189100	651530	323835	152612	6	12	0
MIRD01	GCSL_R53	USA, WA	13/07/07	Oyster	ST-735	cluster_41	101	5191609	651454	324616	152605	6	12	0
MIRE01	GCSL_R54	USA, WA	13/07/07	Oyster	ST-735	cluster_41	96	5189492	651454	333784	158434	6	11	0
MIRF01	GCSL_R55	USA, WA	14/07/07	Oyster	ST-735	cluster_41	100	5191687	651310	323969	152459	6	12	0
MIRG01	GCSL_R56	USA, WA	14/07/07	Oyster	ST-735	cluster_41	101	5191187	651310	324103	152459	6	12	0
14-1072-D-VP	14-1072-D-VP	UK	17/06/14	Oyster	ST-1159	cluster_42	82	5119315	301258	162207	94504	11	22	0
14-1073-H-VP	14-1073-H-VP	UK	17/06/14	Oyster	ST-1159	cluster_42	75	5113814	448042	188616	74204	9	19	0
14-559-B-VP	14-559-B-VP	UK	21/03/14	Oyster	ST-1159	cluster_42	115	5107723	515965	212027	109368	8	17	0
14-692-A-1-VP	14-692-A-1-VP	UK	10/04/14	Oyster	ST-1159	cluster_42	87	5117005	286106	124534	72671	13	26	0
MISL01	GCSL_R144	USA, FL	01/11/07	Oyster	ST-1149	cluster_43	181	5085869	408320	177799	68062	10	21	0
MISM01	GCSL_R145	USA, FL	01/11/07	Oyster	ST-1149	cluster_43	156	5074938	408261	160364	68062	10	22	0
MISN01	GCSL_R146	USA, FL	01/11/07	Oyster	ST-1149	cluster_43	172	5084933	408320	183086	68333	9	20	0
AOOW01	VP766	USA, WA	2007	Plankton	ST--	cluster_44	351	5203969	142435	32385	17899	49	104	46.25
AWLN01	S048	USA	1997	Seafood	ST--	cluster_44	557	5221283	65216	20813	11430	81	166	0
AWLT01	S042	Japan	1984	Clinical	ST-478	cluster_45	735	5115646	57665	14103	7681	112	232	0
AWLU01	S041	China	1993	Clinical	ST-478	cluster_45	710	5090797	65732	14941	8000	106	225	0
AVPW01	VPCR-2010	USA	2010	Water	ST-308	cluster_46	29	6084135	888828	332726	188426	6	12	0
LBHF01	CT4287	ND	2013	Oysters	ST-674	cluster_47	39	5231816	843900	558312	393136	4	7	3.23
MITB01	CDC_K4762	USA, VA	15/08/06	Other	ST-674	cluster_47	108	5103196	871669	392296	264236	5	9	0
MIUF01	CDC_K5323G	USA, VA	ND	Other	ST-674	cluster_47	98	5220531	1367860	322095	179556	4	9	0
MIUG01	CDC_K5323W	USA, VA	ND	Other	ST-674	cluster_47	84	5207450	879874	322199	178767	5	10	0

ID	Alias	Geographical origin	Date	Source	ST	Cluster	No. Contigs	Total length	Largest contig	N50	N75	L50	L75	No. Ns/100 kbp
SRR1118637	CDC_K4762	USA, VA	15/08/06	Other	ST-674	cluster_47	82	5107588	724476	418103	242691	5	9	1.71
LQGX01	A4EZ964	Canada, BC	2004	Clinical	ST-636	cluster_48	49	5101569	739284	407937	189486	5	9	0
MIST01	CDC_K4558W	USA, LA	28/08/06	Wound	ST-636	cluster_48	155	5335587	558903	202283	134208	7	14	0
MISV01	CDC_K4636	USA, NY	25/09/06	Stool	ST-636	cluster_48	138	5340949	558904	208879	137971	7	14	0
MIVD01	CDC_K5618	USA, NY	16/08/07	ND	ST-636	cluster_48	147	5107768	554205	208616	134208	6	13	0
MIVE01	CDC_K5620	USA, NY	23/08/07	ND	ST-636	cluster_48	131	5099609	559459	208616	154293	6	13	0
MIQM01	GCSL_R10	USA, FL	19/03/07	Oyster	ST-313	cluster_49	158	5072012	402843	191541	90174	10	19	0
MISO01	GCSL_R149	USA, FL	19/03/07	Oyster	ST-313	cluster_49	152	5067880	404555	198940	90174	9	18	0
MISP01	GCSL_R150	USA, FL	19/03/07	Oyster	ST-313	cluster_49	149	5066299	405772	191682	90524	10	19	0
AWHK01	S170	China	2007	Environmental	ST-419	cluster_5	377	5062724	124926	35762	19161	45	91	0
AWHS01	S161	China	2006	Seafood	ST-419	cluster_5	480	5061665	80337	24446	13257	70	141	0
AWHV01	S158	China	2006	Seafood	ST-419	cluster_5	497	5062561	93061	23944	12325	71	144	0
AWHX01	S156	China	2006	Seafood	ST-419	cluster_5	584	5060576	80233	17919	9485	93	190	0
AWHY01	S155	China	2006	Seafood	ST-419	cluster_5	577	5059684	80235	19961	10252	82	171	0
AWHZ01	S154	China	2006	Seafood	ST-419	cluster_5	687	5022972	59592	15518	8966	102	206	0
AWIA01	S153	China	2006	Seafood	ST-419	cluster_5	592	5060819	78845	18172	9361	88	185	0
AWIC01	S151	China	2006	Seafood	ST-419	cluster_5	588	5060611	70580	18813	9941	88	180	0
AWID01	S150	China	2006	Seafood	ST-419	cluster_5	592	5060320	63525	18544	9397	87	181	0
AWII01	S144	China	2006	Seafood	ST-419	cluster_5	474	5063512	80231	27181	14264	65	130	0
AWIJ01	S143	China	2006	Seafood	ST-419	cluster_5	461	5060020	134896	25903	12887	63	132	0
AWMK01	S025	China	1992	Clinical	ST-1015	cluster_50	356	5175629	156630	34958	20596	48	95	0
AWMS01	S017	China	1992	Clinical	ST-1015	cluster_50	345	5175677	164509	36112	21565	45	92	0
CFSAN045068_118	CFSAN045068-118	Spain, Galicia	10-2015	Clinic	ST-1031	cluster_51	66	5162986	536041	267801	118175	7	14	0
CFSAN045069_119	CFSAN045069-119	Spain, Galicia	10-2015	Clinic	ST-1031	cluster_51	72	5164650	450308	251676	109652	8	15	0
CFSAN056088	CFSAN056088	Spain, Galicia	09-2016	Clinic	ST-1031	cluster_51	106	5159909	374492	144684	65669	12	25	0
G33	G33	Spain, Galicia	2012	Clinic	ST-1031	cluster_51	42	5444465	697179	289349	163405	7	13	1.01
MIRL01	GCSL_R63	USA, ME	23/07/07	Oyster	ST--	cluster_52	102	5125149	645392	347208	161080	5	11	0
SRR1118652	GCSL_R130	USA, FL	01/10/07	Oyster	ST-1140	cluster_52	107	5062395	627749	334430	163058	6	12	1.98
30824	30824	Spain, Galicia	1999	Clinic	ST-17	cluster_53	56	5083109	488261	203558	112404	9	18	0
428-00	428-00	Spain, Galicia	1998	Clinic	ST-17	cluster_53	66	5138507	320319	145778	93840	12	23	0
AWMI01	S027	USA	2006	Clinical	ST-17	cluster_53	467	5113780	90580	20530	11504	73	153	0
AWMJ01	S026	Thailand	2006	Clinical	ST-17	cluster_53	439	5061731	111831	22512	12195	67	143	0
LHAU01	CFSAN018752	Spain	1998	Stools	ST-17	cluster_53	66	5138507	320319	145778	93840	12	23	0
LHAV01	CFSAN018753	Spain	1999	Stools	ST-17	cluster_53	61	5075961	488256	185164	97262	10	20	0
V06-002	NCTC11344	UK-Maidstone	1980	Clinical	ST-17	cluster_53	123	5043101	293215	97225	57415	16	33	0
AWIU01	S132	China	2005	Clinical	ST-332	cluster_54	421	5104875	119148	28517	15084	57	118	0

ID	Alias	Geographical origin	Date	Source	ST	Cluster	No. Contigs	Total length	Largest contig	N50	N75	L50	L75	No. Ns/100 kbp
AWKS01	S073	Thailand	1997	Clinical	ST-332	cluster_54	530	5059925	70808	23605	11888	67	140	0
AWMH01	S028	Thailand	ND	Clinical	ST-332	cluster_54	443	5074226	126840	32572	17432	44	94	0
AXNN01	VIP4-0434	Hong Kong	2008	Stool	ST-332	cluster_54	75	5061230	581899	375030	128962	6	12	0
LQCW01	C140	Canada, BC	2008	Clinical	ST-332	cluster_54	29	5046146	1126474	636695	313577	3	6	0
AQPJ01	v110	Hong Kong	01/03/10	Shrimp	ST--	cluster_55	366	5426513	546125	143484	77264	11	25	0
MISY01	CDC_K4638	USA, NY	25/09/06	Stool	ST-809	cluster_55	96	5077332	636854	248530	169819	6	12	0
AWLM01	S049	Japan	1984	Clinical	ST--	cluster_56	714	5229483	52661	16173	8336	99	206	0
AWLZ01	S036	Thailand	ND	Clinical	ST-8	cluster_56	498	5033625	89010	20685	12329	79	156	0
AWMA01	S035	Japan	1984	Clinical	ST-8	cluster_56	543	5120372	92669	21594	11911	71	150	0
AWMB01	S034	India	1999	Clinical	ST-8	cluster_56	460	5148063	103853	25656	15083	62	128	0
AWMC01	S033	China	1994	Clinical	ST-8	cluster_56	477	5171831	104100	29792	15383	54	113	0
AWMD01	S032	Philippines	1998	Clinical	ST-1016	cluster_56	767	5117989	57933	13893	7453	117	241	0
AWMN01	S022	Japan	1984	Clinical	ST-8	cluster_56	444	5222979	120859	32171	17612	51	105	0
LRFV01	F30368	Canada, BC	2006	Clinical	ST-8	cluster_56	30	5090449	815154	524972	353243	4	7	0
LRSV01	07-2964	Canada, SK	2007	Clinical	ST-8	cluster_56	32	5120702	743040	415597	232340	5	8	0
AWJC01	S123	Japan	1984	Clinical	ST-217	cluster_57	748	5070876	57600	12246	6939	121	256	0
AWJD01	S122	India	1999	Clinical	ST-217	cluster_57	738	5087743	50642	12750	7043	120	252	0
LFZA01	S439-9	Canada	01/07/12	Oyster	ST-1155	cluster_58	208	6052164	752543	149287	58890	10	26	0
MIQY01	GCSL_R42	USA, WA	26/07/07	Oyster	ST-1155	cluster_58	105	4981892	602722	314803	118402	6	12	0
AVOL01	3259	USA	2007	Stool	ST-479	cluster_59	6	5360869	3370862	3370862	759672	1	2	0
AZMN01	EKP-008	Bangladesh	2007	Water	ST-479	cluster_59	323	5282636	514908	100792	27228	11	38	0
605.fsa_nt	605	USA	2007	Environmental	ST-3	cluster_6	120	5153347	557945	241706	138524	8	15	0
906-97	906-97	Peru	1996	Clinical	ST-3	cluster_6	50	5100874	615203	427204	170794	5	9	0
9808-1	9808-1	Spain, Galicia	2004	Clinical	ST-3	cluster_6	60	5073596	536487	267687	125198	7	14	0
ACFM01	Peru-466	Peru	1996	Clinical	ST-3	cluster_6	149	5037488	273858	81497	38043	19	43	0
ACFO01	AN-5034	Bangladesh	1998	Clinical	ST-3	cluster_6	54	5199902	1183081	346246	116797	5	11	0
ACKB01	K5030	India	2005	Clinical	ST-3	cluster_6	164	5028244	657114	62978	34161	18	46	0
AN-16000_ST3	AN-16000	Bangladesh	1998	Clinical	ST-3	cluster_6	54	5124055	691206	395011	153223	5	11	0
AN-2189_ST3	AN-2189	Bangladesh	1998	Clinical	ST-3	cluster_6	49	5112259	559573	312334	157889	6	11	0
AN-5034_ST3	AN-5034	Bangladesh	1998	Clinical	ST-3	cluster_6	56	5159741	685664	312403	159474	6	11	0
AN-8373_ST3	AN-8373	Bangladesh	1998	Clinical	ST-3	cluster_6	60	5085312	580137	288679	153383	6	12	0
AO-24491_ST3	AO-24491	Bangladesh	1999	Clinical	ST-3	cluster_6	54	5156258	845551	394558	168156	5	9	2.08
AOOV01	VP551	USA, WA	2007	Water	ST-3	cluster_6	30	5226872	885804	712378	431768	4	6	0.61
AOOY01	863	USA, WA	2007	Plankton	ST-3	cluster_6	37	5226554	1265784	431743	301124	4	7	0.59
AOOZ01	930	USA, WA	2007	Oyster	ST-3	cluster_6	12	5165306	1420642	699136	301119	3	5	0

ID	Alias	Geographical origin	Date	Source	ST	Cluster	No. Contigs	Total length	Largest contig	N50	N75	L50	L75	No. Ns/100 kbp
AP-11243_ST51	AP-11243	Bangladesh	2000	Clinical	ST51/ST-3	cluster_6	53	5116032	563931	312374	137615	6	13	0
AVOJ01	VP232	India	1998	Stool	ST-3	cluster_6	52	5123959	576321	171658	80530	7	19	0
AVOK01	VP250	India	1998	Stool	ST-3	cluster_6	5	5202656	3317379	3317379	907448	1	2	0
AVOM01	NIHCB0603	Bangladesh	2006	Stool	ST-3	cluster_6	13	5225215	1438315	1292484	346446	2	5	0
AVON01	VP-NY4	India	1997	Stool	ST-3	cluster_6	14	5247801	1718398	1120023	331543	2	5	0
AVPV01	949	USA	2006	Oyster	ST-3	cluster_6	7	5179240	1398079	972111	957879	3	4	0
AWIO01	S138	China	2007	Clinical	ST-3	cluster_6	500	5133647	99135	22106	11747	76	157	0
AWIP01	S137	China	2005	Clinical	ST-3	cluster_6	708	5015616	60156	15592	8294	96	204	0
AWIQ01	S136	China	2004	Clinical	ST-3	cluster_6	490	5065064	119154	21577	12492	71	148	0
AWIR01	S135	China	2003	Clinical	ST-3	cluster_6	434	5058833	103139	25931	15031	66	129	0
AWIT01	S133	China	2005	Clinical	ST-3	cluster_6	513	5046254	108377	22687	13673	68	139	0
AWIV01	S131	ND	2003	Clinical	ST-3	cluster_6	434	5132298	108359	26782	14538	59	122	0
AWIZ01	S126	China	ND	Clinical	ST-3	cluster_6	427	5064103	119159	27924	14320	57	119	0
AWKB01	S094	Thailand	1996	Clinical	ST-3	cluster_6	504	5057048	96686	22106	11891	67	143	0
AWKC01	S093	Japan	1998	Clinical	ST-3	cluster_6	481	4953111	113681	29016	14340	53	114	0
AWKD01	S092	China	1996	Clinical	ST-3	cluster_6	463	5164347	87681	28070	14968	60	124	0
AWKE01	S091	India	1999	Clinical	ST-3	cluster_6	753	5138592	69050	14759	7692	101	220	0
AWKF01	S090	China	1999	Clinical	ST--	cluster_6	734	5040869	57208	16448	9356	92	191	0
AWKG01	S088	Singapore	1998	Clinical	ST-3	cluster_6	405	5079007	99760	30513	17612	49	104	0
AWKH01	S087	Singapore	1998	Clinical	ST--	cluster_6	449	5080366	99328	27007	14737	56	119	0
AWKI01	S086	Thailand	1999	Clinical	ST-3	cluster_6	469	5066586	86005	25865	13114	60	128	0
AWKJ01	S083	Japan	1998	Clinical	ST-3	cluster_6	442	5111344	92923	28639	15384	59	118	0
AWKK01	S082	Thailand	ND	Clinical	ST--	cluster_6	555	4998104	65795	18688	10575	79	167	0
AWKL01	S081	South Korea	ND	Clinical	ST-3	cluster_6	493	5058310	98462	22930	12662	66	140	0
AWKM01	S079	Indonesia	ND	Clinical	ST-3	cluster_6	664	5113397	58687	16129	8741	103	210	0
AWKN01	S078	China	1999	Clinical	ST-3	cluster_6	467	5113590	107407	23501	14114	66	136	0
AWKO01	S077	China	1999	Clinical	ST-3	cluster_6	554	5113464	75863	18729	11142	87	174	0
AWKP01	S076	China	1999	Clinical	ST-3	cluster_6	449	5112235	127452	25100	14463	64	129	0
AWKQ01	S075	China	1999	Clinical	ST-3	cluster_6	721	5114211	53034	14151	7568	114	238	0
AWKR01	S074	China	1997	Clinical	ST-3	cluster_6	689	5138812	53305	14150	7781	113	233	0
AWKT01	S072	Bangladesh	1998	Clinical	ST-3	cluster_6	433	5075541	107390	27675	15319	55	116	0
AWKU01	S071	Bangladesh	1998	Clinical	ST-3	cluster_6	424	5084519	95178	28640	16074	53	114	0
AWKV01	S070	Thailand	ND	Clinical	ST--	cluster_6	630	5052130	65110	15138	8992	107	212	0
AWKW01	S069	Thailand	ND	Clinical	ST--	cluster_6	414	5053124	102347	28922	14887	55	115	0
AWKX01	S068	China	1997	Clinical	ST-3	cluster_6	529	5133117	69201	21152	12197	77	157	0

ID	Alias	Geographical origin	Date	Source	ST	Cluster	No. Contigs	Total length	Largest contig	N50	N75	L50	L75	No. Ns/100 kbp
AWKY01	S067	China	1997	Clinical	ST-3	cluster_6	513	5064703	82712	21213	11962	72	151	0
AWKZ01	S066	China	1997	Clinical	ST-3	cluster_6	626	5057879	69663	16046	8889	95	200	0
AWLA01	S065	China	1998	Clinical	ST-3	cluster_6	509	5058701	87215	21258	12199	76	154	0
AWLB01	S064	China	1998	Clinical	ST-3	cluster_6	677	5066603	70355	14862	8305	99	209	0
AWLC01	S063	China	1998	Clinical	ST-3	cluster_6	525	5129893	74447	22691	12340	73	147	0
AWLD01	S062	Singapore	1998	Clinical	ST-3	cluster_6	454	5063284	110779	22532	13319	67	138	0
AXNJ01	VIP4-0395	Hong Kong	2007	Stool	ST-3	cluster_6	86	5120046	492089	232863	137643	8	14	0.02
AXNK01	VIP4-0439	Hong Kong	2008	Stool	ST-3	cluster_6	90	5137870	492075	188126	100365	9	18	0.02
AXNL01	VIP4-0445	Hong Kong	2008	Stool	ST--	cluster_6	908	4966579	41619	9963	5201	153	322	0.28
AXNM01	VIP4-0407	Hong Kong	2008	Stool	ST-3	cluster_6	91	5096056	468296	235555	127262	8	15	0.02
AZGU01	605	USA	2006	Water	ST-3	cluster_6	119	5147903	557945	241706	138524	8	15	0
AZGV01	861	USA	2006	Water	ST-3	cluster_6	239	5162308	174170	53624	30285	30	62	0
AZKN01	B-265	Mozambique	2004	Stool	ST-3	cluster_6	109	5184899	377503	260673	116102	8	16	0
AZNA01	EKP-021	Bangladesh	2008	Water	ST-3	cluster_6	759	5133013	69465	13199	6860	112	251	0
BAC-98-3372	BAC-98-3372	USA	1998	Clinical	ST-3	cluster_6	62	5091727	655276	288853	137371	6	12	0
BAC-98-3374	BAC-98-3374	USA	1998	Clinical	ST-3	cluster_6	73	5180743	558511	312752	100423	7	14	0
BAC-98-4092_ST3	BAC-98-4092	USA	1998	Clinical	ST-3	cluster_6	59	5179914	691433	387108	138945	6	12	0
CFSAN056086	CFSAN056086	Spain, Galicia	08-2016	Clinical	ST-3	cluster_6	63	5110254	527820	199151	122979	7	15	0
JABV01	EKP-026	Bangladesh	2008	Water	ST-3	cluster_6	196	5100902	282349	71441	39879	21	45	0
JACH01	EKP-028	Bangladesh	2008	Water	ST-3	cluster_6	875	5096768	36078	10548	5888	157	314	0
JAHD01	IDH02189	India	2009	Stool	ST-3	cluster_6	162	5092302	284252	107900	54676	16	32	0
JAIH01	IDH02640	India	2009	Stool	ST-3	cluster_6	230	5203790	181213	60652	33765	26	54	0
JAII01	Peru-288	Peru	2001	Stool	ST-3	cluster_6	142	5180217	500261	168272	72778	10	22	0
JALG01	V14/01	Chile	2001	Stool	ST-3	cluster_6	142	5254370	345301	159713	83363	12	22	0
JALH01	V-223/04	Chile	2004	Stool	ST--	cluster_6	5120	6322739	36117	2396	1274	631	1471	0
JDFK01	VP-48	India	1996	Stool	ST--	cluster_6	231	5108901	189871	55635	33903	29	58	0
JMMS01	AF91	USA, FL	2006	Sediment	ST-1517	cluster_6	111	5176263	728768	269871	134312	6	13	0.37
JNTG02	CFSAN007449	USA, MD	21/08/12	Stool	ST-3	cluster_6	55	5119321	653511	346912	159791	5	11	0
JNTH02	CFSAN007450	USA, MD	22/08/12	Stool	ST-3	cluster_6	65	5114004	680205	288413	105851	6	14	0
JNTI02	CFSAN007451	USA, MD	24/08/12	Stool	ST-3	cluster_6	49	5121158	844540	401590	218410	5	9	0
JYJS01	10-4251	Canada, BC	2006	Clinical	ST-3	cluster_6	155	5219165	654860	157866	54280	9	24	0
JYNG01	04-2549	Canada, ON	2004	Clinical	ST-3	cluster_6	138	5129237	557727	411035	158101	5	11	0
JZAR01	09-4435	Canada, BC	2009	Clinical	ST-3	cluster_6	115	5120353	652955	288608	148967	6	12	0
KXV-641_ST3	KXV-641	Japan	1998	Clinical	ST-3	cluster_6	51	5094220	653581	394740	168115	5	10	0
LASF01	04-2551	Canada, ON	2004	Clinical	ST-3	cluster_6	85	5187612	654382	339951	169754	6	11	0
LASG01	07-1339	Canada, BC	2007	Clinical	ST-3	cluster_6	88	5251833	844096	373794	158770	5	10	0

ID	Alias	Geographical origin	Date	Source	ST	Cluster	No. Contigs	Total length	Largest contig	N50	N75	L50	L75	No. Ns/100 kbp
LFUJ01	PMC58.5	Chile, PM	2005	Feces	ST-3	cluster_6	14	5154023	1359266	1090964	550307	3	4	26.89
LFUK01	PMA109.5	Chile, PM	2005	Environmental	ST--	cluster_6	20	5338346	1262094	582970	451237	3	6	50.71
LFUL01	PMA37.5	Chile, PM	2005	Environmental	ST-3	cluster_6	94	5096624	338698	175000	65564	11	24	0
LFUM01	PMC58.7	Chile, PM	2007	Feces	ST-3	cluster_6	23	5163166	998714	547261	264751	4	7	45.36
LFUN01	ATC210	Chile, Antofagasta	1998	Feces	ST-3	cluster_6	23	5136970	1003139	838297	263071	3	6	28.35
LFUO01	PMC14.7	Chile, PM	2007	Feces	ST-3	cluster_6	23	5133772	1101814	853734	346830	3	6	48.54
LFUP01	PMC48	Chile, PM	2004	Feces	ST-3	cluster_6	13	5138290	1406770	992458	550693	3	4	60.58
LFUQ01	ATC220	Chile, Antofagasta	1998	Feces	ST-3	cluster_6	19	5145131	1045440	547874	479769	4	6	15
LFUR01	RIMD-2210633	Japan	1996	Feces	ST-3	cluster_6	17	5149386	1099114	619178	442579	3	6	25.81
LHAW01	CFSAN018754	Spain	2004	Stools	ST-3	cluster_6	60	5073596	536487	267687	125198	7	14	0
LHAZ01	CFSAN018757	Peru	1997	Stools	ST-3	cluster_6	72	5099401	505725	288855	123233	7	14	0
LOHN01	A4EZ927	Canada, BC	2004	Clinical	ST-3	cluster_6	48	5104589	653730	373935	168962	5	11	0
LPZS01	Gxw_7004	China, Guangxi	09/06/07	Stool	ST-3	cluster_6	65	5158042	885831	323009	243703	5	10	7.04
LQCD01	A5Z273	Canada, BC	2005	Clinical	ST--	cluster_6	44	5098054	653249	373921	242770	5	10	0
LQCF01	A5Z853	Canada, BC	2005	Clinical	ST-3	cluster_6	41	5142950	653052	427483	189215	5	10	0
LRFS01	A3EZ136	Canada, BC	2003	Clinical	ST-3	cluster_6	45	5089443	843218	373928	168017	5	10	0
LRFW01	F63267	Canada, BC	2006	Clinical	ST-3	cluster_6	47	5121492	653996	427352	189105	5	10	0
MDTV01	Klin	Sweden	2010	Stool	ST-3	cluster_6	56	5104502	580121	260007	159750	7	12	0
MISW01	CDC_K4637G	USA, NY	01/10/06	Stool	ST-3	cluster_6	150	5163379	652904	259160	105339	6	14	0
MISX01	CDC_K4637W	USA, NY	01/10/06	Stool	ST-3	cluster_6	133	5156414	652523	268238	122321	6	13	0
MITF01	CDC_K4775	USA, GA	24/02/07	Stool	ST-3	cluster_6	121	5158365	642942	312581	105282	6	12	0
MITN01	CDC_K5010G	USA, MA	16/09/06	Stool	ST-3	cluster_6	146	5122961	652927	242331	108117	7	14	0
MITO01	CDC_K5010W	USA, MA	16/09/06	Stool	ST-3	cluster_6	133	5118603	652589	259047	158268	7	13	0
MITP01	CDC_K5058	USA, TX	15/05/07	Stool	ST-3	cluster_6	84	5114568	840257	301866	118359	5	11	0
MIVA01	CDC_K5528	USA, GA	06/10/07	Stool	ST-3	cluster_6	108	5101848	652922	275983	158209	6	12	0
N314	N314	Spain, Galicia	07/2016	Clinic	ST-3	cluster_6	61	5073531	691322	347447	118524	5	12	0
P682-Peru	P682	Peru	2009	Shellfish	ST-3	cluster_6	109	5090692	461903	157162	72043	11	24	0
P729-Peru	P729	Peru	2009	Shellfish	ST-3	cluster_6	94	5094250	424608	157889	83086	12	24	0
P860	P860	Peru	2009	Shellfish	ST-3	cluster_6	147	5084286	255037	95820	54325	19	36	0
PMA-109-5_ST3	PMA-109-5	Chile	2005	Environmental	ST-3	cluster_6	95	5216802	542580	166936	80001	9	22	0
PMA-37-5_ST3	PMA-37-5	Chile	2005	Environmental	ST-3	cluster_6	63	5101408	615044	288855	108221	6	13	0
SRR1013448	NY-4	ND	ND	ND	ST-3	cluster_6	1531	4733387	34450	6569	3209	191	449	19.44
SRR1013449	NY-4	ND	ND	ND	ST-3	cluster_6	1576	4758247	46537	6445	3025	203	468	19.66
SRR1013450	NY-4	ND	ND	ND	ST-3	cluster_6	1887	4563130	31958	4554	2226	277	623	21.4

ID	Alias	Geographical origin	Date	Source	ST	Cluster	No. Contigs	Total length	Largest contig	N50	N75	L50	L75	No. Ns/100 kbp
SRR1013451	NY-4	ND	ND	ND	ST--	cluster_6	1523	4796425	59155	6266	2942	216	494	16.91
SRR1013452	NY-4.pexsA	ND	ND	ND	ST-3	cluster_6	1369	4939359	59754	7194	3733	195	429	16.99
SRR1013453	NY-4.pexsA	ND	ND	ND	ST-3	cluster_6	1356	4892548	51817	7312	3508	185	419	18.25
SRR1013454	NY-4.pexsD	ND	ND	ND	ST-3	cluster_6	1429	4851572	51841	7300	3421	187	425	17.28
SRR1013455	NY-4.pexsD	ND	ND	ND	ST-3	cluster_6	1631	4728723	62417	5944	2784	214	501	18.22
SRR1013456	NY-4	ND	ND	ND	ST--	cluster_6	2008	4481678	26828	4047	1993	291	673	33.62
SRR1013457	NY-4	ND	ND	ND	ST--	cluster_6	1872	4648201	59449	4608	2312	261	603	23.32
TX2103_ST3	TX2103	USA	1998	Clinical	ST-3	cluster_6	58	5151098	691360	347728	157888	5	11	0
V12-024	V12-024	UK	23/08/14	Pandemic	ST-3	cluster_6	84	5103231	461944	125273	69379	12	25	0
Vp155_ST3	Vp155	India	1996	Clinical	ST-3	cluster_6	46	5099037	614021	427397	170747	5	10	0
VP16	VP16	USA, MD	21/08/12	Stool	ST-3	cluster_6	55	5119321	653511	346912	159791	5	11	0
VP17	VP17	USA, MD	22/08/12	Stool	ST-3	cluster_6	58	5117310	542574	269751	153408	7	13	0
VP18_ST3	VP18	USA, MD	24/08/12	Stool	ST-3	cluster_6	49	5121158	844540	401590	218410	5	9	0
Vp208_ST3	Vp208	India	1997	Clinical	ST-3	cluster_6	52	5146731	579916	318441	159527	6	11	0
Vp2_ST27	Vp2	Korea	1998	ND	ST-3	cluster_6	38	5100433	691202	505713	288853	5	8	0
VP53	VP53	China	2005	Clinical	ST-3	cluster_6	40	5138114	580072	401743	228956	6	9	0
Vp81_ST3	Vp81	India	1996	Clinical	ST-3	cluster_6	53	5089891	691202	305818	243394	6	10	0
Vp96_ST3	Vp96	India	1996	Clinical	ST-3	cluster_6	47	5092377	627962	427240	168797	5	9	0
VpHY145_ST3	VpHY145	Thailand	1999	Clinical	ST-3	cluster_6	50	5149473	691202	312515	159519	6	11	0
VpHY191_ST3	VpHY191	Thailand	1999	Clinical	ST-3	cluster_6	51	5154847	627791	318072	153020	5	12	0
JYJR01	10-4244	Canada, BC	2006	Clinical	ST-141	cluster_60	128	5268926	903551	383602	244419	5	9	0
LASM01	10-4243	Canada, BC	2006	Clinical	ST-141	cluster_60	76	5254404	1799250	525588	461169	3	5	0
LODO01	A4EZ703	Canada, BC	2004	Clinical	ST-141	cluster_60	34	5146304	1799251	525437	409722	3	5	0
AWJW01	S100	USA	1990	Seafood	ST-324	cluster_61	508	5025482	101922	21352	10499	74	158	0
AZGT01	50	USA	2006	Stool	ST-34	cluster_61	107	5057723	567190	285062	110719	6	13	0
JNUE01	98-513-F52	USA, LA	1998	Gulf Coast isolate	ST-34	cluster_61	120	5048410	357713	126064	75429	11	24	0
JNUM02	CFSAN007458	USA, MD	2010	Oyster	ST-34	cluster_61	52	5061948	729103	321455	152086	6	11	0
JWSR01	98-513-F52	USA, LA	1998	Environmental	ST-34	cluster_61	4	5173738	3055020	3055020	1817167	1	2	0
LFZF01	S440-7	Canada	01/07/12	Oyster	ST-34	cluster_61	111	5138777	1062149	470844	215371	4	8	0
LIRQ01	S372-5	Canada	01/06/11	Oyster	ST-324	cluster_61	81	5147783	658515	480510	214140	5	9	0
LRFR01	A2EZ743	Canada, BC	2002	Clinical	ST-324	cluster_61	34	5063270	1071346	526416	269300	4	7	0
MIQP01	GCSL_R16	USA, FL	30/04/07	Oyster	ST-34	cluster_61	114	5051817	567077	284110	142504	6	12	0
MIUD01	CDC_K5306	USA, GA	23/07/07	Stool	ST-34	cluster_61	117	5045861	611876	314904	133022	5	12	0
OAG95	OAG95	Spain	2007	Environmental	ST--	cluster_62	71	5137448	942522	398612	202837	5	9	0
MIUC01	CDC_K5282	USA, HI	24/05/07	Other	ST--	cluster_63	79	4963427	872931	385892	147718	5	9	0

ID	Alias	Geographical origin	Date	Source	ST	Cluster	No. Contigs	Total length	Largest contig	N50	N75	L50	L75	No. Ns/100 kbp
SRR1118617	CDC_K5282	USA, HI	24/05/07	Other	ST--	cluster_63	69	4968160	881312	408321	167264	5	9	1.13
MITH01	CDC_K4857G	USA, HI	28/01/07	Stool	ST-79	cluster_64	103	5038683	579946	293993	139655	6	12	0
MITI01	CDC_K4857W	USA, HI	28/01/07	Stool	ST-79	cluster_64	108	5039775	579946	302174	138297	6	12	0
PQ110_2nd	PQ110	Spain	2006	Environmental	ST-79	cluster_64	62	5185187	715662	358099	179712	6	11	0
OAG100	OAG100	Spain	2007	Environmental	ST-1121	cluster_65	64	5167508	530824	224692	123336	8	15	0
OAG99	OAG99	Spain	2007	Environmental	ST-1121	cluster_65	63	5174152	683300	270506	149822	7	14	0.56
AWJE01	S121	Thailand	ND	Clinical	ST-934	cluster_66	722	5054264	56701	12282	7069	118	252	0
AWJJ01	S116	Thailand	ND	Clinical	ST-934	cluster_66	777	5081860	56716	11871	6751	124	263	0
BAVG01	TUMSAT_DE2_S2	Thailand	ND	Shrimp	ST-970	cluster_67	96	5623531	919101	266480	103581	8	15	13.62
JPKS01	NCKU_TV_3HP	Thailand	1999	Shrimp	ST-970	cluster_67	100	5543408	699315	266574	155432	7	13	38.65
JPKT01	NCKU_TV_5HP	Thailand	1999	Shrimp	ST-970	cluster_67	82	5534040	699109	266695	148821	7	14	17.1
AAWQ01	AQ3810	Singapore	1983	Clinical	ST-87	cluster_68	1073	5771228	295134	30448	7415	45	133	0
AZJP01	AQ3810	Singapore	1983	Stool	ST--	cluster_68	1152	5013700	33175	7461	4147	197	417	0
MITC01	CDC_K4763	USA, VA	25/08/06	Stool	ST--	cluster_69	129	5287070	660007	355269	137184	6	11	0
MITD01	CDC_K4764D	USA, VA	13/10/06	Stool	ST--	cluster_69	114	5276530	660531	355176	142435	6	11	0
AWJB01	S124	China	1992	Clinical	ST-331	cluster_7	697	5139803	45286	12697	6979	125	258	0
OJL90_2nd	OJL90	Spain	2007	Environmental	ST-331	cluster_7	44	5228725	687741	338449	151455	6	11	0
PH157	PH157	Spain	ND	Environmental	ST-331	cluster_7	39	5234591	853195	470592	201166	5	8	0
V05-002	V05-002	UK-Norfolk	1972	Clinical	ST-331	cluster_7	80	5228043	384624	153338	86627	13	25	0
MITM01	CDC_K5009W	USA, MA	07/08/06	Stool	ST-749	cluster_70	130	5125537	640491	438284	197083	5	10	0
MITU01	CDC_K5125	USA, MS	11/06/07	Other	ST-749	cluster_70	134	5134866	640564	316428	142673	5	12	0
MIQI01	GCSL_R5	USA, TX	14/03/07	Oyster	ST-1133	cluster_71	95	5185921	1075622	460669	171326	4	8	0
MIQJ01	GCSL_R6	USA, TX	14/03/07	Oyster	ST-1133	cluster_71	107	5087598	710832	402719	155559	5	10	0
LFWK01	S383-6	Canada	2011	Seafood	ST-1134	cluster_72	79	5106275	705971	461802	272561	5	8	0
LFWM01	S448-16	Canada	2012	Canada	ST-1134	cluster_72	76	5116547	1270814	523860	273241	4	7	0
LFZB01	S499-7	Canada	01/09/13	Oyster	ST-1134	cluster_72	90	5113926	693890	523860	271438	5	8	0
MIQK01	GCSL_R7	USA, TX	14/03/07	Oyster	ST-1134	cluster_72	138	5052796	641707	342982	166021	5	11	0
MIQL01	GCSL_R8	USA, TX	14/03/07	Oyster	ST-1134	cluster_72	119	5200929	641707	368783	165968	5	11	0
LHAX01	CFSAN018755	Spain	2002	Missing	ST-52	cluster_73	76	5112517	955685	379292	132019	5	11	0
UCM-V441	UCM-V441	Spain	2002	Environmental	ST-52	cluster_73	62	5114969	874048	429329	187020	4	9	0
MITQ01	CDC_K5059G	USA, TX	10/05/07	Other	ST-1147	cluster_74	80	4959751	887328	467008	179356	4	8	0
MITR01	CDC_K5059W	USA, TX	10/05/07	Other	ST-1147	cluster_74	123	4953954	887363	466965	179068	4	8	0
LHAY01	CFSAN018756	Spain	2003	Missing	ST--	cluster_75	64	4969236	645827	246923	122314	6	12	0
UCM-V586	UCM-V586	Spain	2003	Environmental	ST--	cluster_75	48	4973439	645837	404185	208545	5	9	5.17
BAVF01	TUMSAT_DE1_S1	Thailand	ND	Shrimp	ST-114	cluster_76	127	5280365	650884	314261	152913	6	11	61.53
JPKU01	NCKU_CV_CHN	China	2010	Shrimp	ST-114	cluster_76	144	5318783	915654	551331	255347	4	8	21.5

ID	Alias	Geographical origin	Date	Source	ST	Cluster	No. Contigs	Total length	Largest contig	N50	N75	L50	L75	No. Ns/100 kbp
LPVL01	C146	Canada, BC	2008	Clinical	ST-1060	cluster_77	51	5212184	1200282	404244	169590	4	9	0
LRTG01	A3EZ936	Canada, BC	2003	Clinical	ST-1060	cluster_77	51	5245252	812388	387604	196311	5	9	0
LHBG01	CFSAN018764	USA	2004	Missing	ST-58	cluster_78	46	5139686	1090046	642262	177713	3	9	0.56
LHBH01	CFSAN018765	USA	2004	Missing	ST-58	cluster_78	60	5245157	935300	256190	155157	6	12	0
MITG01	CDC_K4842	USA, MD	16/10/06	Stool	ST-1144	cluster_79	115	5194684	922863	322967	142530	5	12	0
SRR1118634	CDC_K4842	USA, MD	16/10/06	Stool	ST-1144	cluster_79	89	5204948	931137	384547	179442	4	9	2.37
AVOI01	VP2007-095	USA	2007	Stool	ST-631	cluster_8	21	5175863	710002	485232	286686	5	8	0
JNSM01	CFSAN007429	USA, MD	15/06/12	Stool	ST--	cluster_8	285	5033496	141406	41218	21619	38	80	0
JNSN01	CFSAN007430	USA, MD	12/07/12	Stool	ST--	cluster_8	298	5068114	141322	36375	23363	42	86	0
JNSO01	CFSAN007431	USA, MD	17/07/12	Stool	ST-631	cluster_8	278	5090038	187280	44754	24357	34	72	0
JNSP01	CFSAN007432	USA, MD	16/06/13	Stool	ST-631	cluster_8	273	5097898	160669	44908	24380	36	75	0
JNSQ01	CFSAN007433	USA, MD	11/07/13	Stool	ST--	cluster_8	319	5214691	156370	34995	22005	45	91	0
JNSR01	CFSAN007434	USA, MD	17/07/13	Stool	ST--	cluster_8	233	5109515	175851	59970	37501	28	54	0
JNSS01	CFSAN007435	USA, MD	03/08/13	Stool	ST--	cluster_8	223	5069192	189785	61231	31726	28	57	0
JNST01	CFSAN007436	USA, MD	09/08/13	Stool	ST-631	cluster_8	214	5085334	233102	65831	37652	27	53	0
LBHP01	MAVP-E	USA, MA	2010	Clinical	ST-631	cluster_8	246	5285309	684930	339816	183046	6	11	8.57
LFZE01	S487-4	Canada	01/08/13	Oyster	ST-631	cluster_8	102	5181575	867905	431578	375930	5	8	0
LRAJ01	09-4436	Canada, PEI	2009	Clinical	ST-631	cluster_8	41	5151693	700330	431727	187598	5	9	0
MITW01	CDC_K5276	USA, NY	20/04/07	Stool	ST-631	cluster_8	136	5168475	635177	339585	146274	6	11	0
MIVC01	CDC_K5582	USA, GA	10/10/07	Stool	ST-631	cluster_8	104	5174332	684657	339585	186500	6	10	0
MIRK01	GCSL_R62	USA, ME	23/07/07	Oyster	ST-1136	cluster_80	126	5176254	565199	192245	82200	7	18	0
MITE01	CDC_K4764L	USA, VA	13/10/06	Stool	ST-1156	cluster_81	111	5033430	895696	519873	195271	4	9	0
AWHN01	S166	China	2007	Environmental	ST-1011	cluster_82	522	5005906	109549	23056	11489	70	147	0
AWMM01	S023	China	1994	Clinical	ST-610	cluster_83	685	5123331	79832	15161	8907	106	215	0
AXNS01	VIP4-0447	Hong Kong	2008	Oyster	ST-396	cluster_84	113	5367084	668073	305898	110106	6	14	0
AWHI01	S172	China	2007	Environmental	ST-550	cluster_85	363	5051952	138365	37669	21486	41	83	0
AMRZ01	SNUVpS-1	South Korea	31/05/09	Seafood	ST-917	cluster_86	60	5241845	663644	237357	135456	7	13	0
AWMQ01	S019	USA	1998	Clinical	ST-563	cluster_87	466	5079255	79435	23909	12593	72	144	0
AWHR01	S162	Thailand	2007	Seafood	ST-595	cluster_88	513	5040912	69197	20676	11301	78	161	0
MIQT01	GCSL_R29	USA, FL	27/05/07	Oyster	ST-734	cluster_89	104	4946371	842854	295296	188976	6	11	0
AWIS01	S134	China	2005	Clinical	ST-527	cluster_9	459	5189192	84812	27182	14744	60	124	0
AWIW01	S130	ND	2003	Clinical	ST-527	cluster_9	445	5190597	83271	26145	15083	63	129	0
AWLG01	S057	China	1994	Clinical	ST-69	cluster_9	606	5132316	92894	20175	11136	75	158	0
AWLH01	S056	China	1994	Clinical	ST--	cluster_9	865	5264929	55094	12396	7194	123	260	0
AWLI01	S055	China	1994	Clinical	ST-69	cluster_9	448	5052163	142510	35392	19677	42	87	0
AWLJ01	S054	Thailand	ND	Clinical	ST-69	cluster_9	684	5180948	73277	14490	8635	104	218	0

ID	Alias	Geographical origin	Date	Source	ST	Cluster	No. Contigs	Total length	Largest contig	N50	N75	L50	L75	No. Ns/100 kbp
MITK01	CDC_K4859	USA, HI	15/02/07	Other	ST--	cluster_90	102	5179426	917103	238239	142635	6	13	0
LHBA01	CFSAN018758	Peru	1999	Stools	ST-19	cluster_91	64	5360551	605324	261361	142941	7	13	0
JNTB01	CFSAN007444	USA, MD	25/06/12	Wound	ST-677	cluster_92	135	4952823	386344	123984	69186	13	27	0
LRAH01	04-2550	Canada, ON	2004	Clinical	ST-630	cluster_93	40	5099458	1046942	537513	180855	4	8	0
AWHL01	S168	China	2007	Environmental	ST-627	cluster_94	349	5028507	127693	39979	22677	39	79	0
AXNO01	VIP4-0430	Hong Kong	2008	Oyster	ST-507	cluster_95	1009	5564125	706124	281842	104591	6	13	0.08
LFYN01	S357-21	Canada	2010	Oyster	ST-102	cluster_96	153	5074321	380087	173413	106388	10	19	0
AWHG01	S174	China	2007	Environmental	ST-1012	cluster_97	421	5013146	98821	37577	22315	43	86	0
AWMG01	S029	Spain	1981	Environmental	ST-810	cluster_98	594	4974353	105136	24807	12889	64	131	0
LQGU01	K23	India	09/05/13	Seafood	ST-1052	cluster_99								

*Strains are grouped by sequence type; sequence types are separated by color. Location includes state, province, or region, if known. ID, identifier; ND, not determined; ST, sequence type; ST--, new sequence type with $\geq$1 new variant allele.
